# Limited effect of thermal pruning on wild blueberry crop and its root-associated microbiota

**DOI:** 10.3389/fpls.2022.954935

**Published:** 2022-08-04

**Authors:** Simon Morvan, Maxime C. Paré, Anne Schmitt, Jean Lafond, Mohamed Hijri

**Affiliations:** ^1^Institut de Recherche en Biologie Vègétale, Département de sciences biologiques, Université de Montréal, Montréal, QC, Canada; ^2^Laboratoire sur les écosystèmes boréaux terrestres (EcoTer), Département des Sciences Fondamentales, Université du Québec à Chicoutimi, Chicoutimi, QC, Canada; ^3^Direction générale des sciences et de la technologie, Agriculture et Agroalimentaire Canada, Gouvernement du Canada, Normandin, QC, Canada; ^4^African Genome Center, Mohammed VI Polytechnic University, Ben Guerir, Morocco

**Keywords:** thermal pruning, wild blueberry, *Vaccinium angustifolium* Ait., microbial community, ericoid mycorrhiza, amplicon sequencing

## Abstract

Thermal pruning was a common pruning method in the past but has progressively been replaced by mechanical pruning for economic reasons. Both practices are known to enhance and maintain high yields; however, thermal pruning was documented to have an additional sanitation effect by reducing weeds and fungal diseases outbreaks. Nevertheless, there is no clear consensus on the optimal fire intensity required to observe these outcomes. Furthermore, fire is known to alter the soil microbiome as it impacts the soil organic layer and chemistry. Thus far, no study has investigated into the effect of thermal pruning intensity on the wild blueberry microbiome in agricultural settings. This project aimed to document the effects of four gradual thermal pruning intensities on the wild blueberry performance, weeds, diseases, as well as the rhizosphere fungal and bacterial communities. A field trial was conducted using a block design where agronomic variables were documented throughout the 2-year growing period. MiSeq amplicon sequencing was used to determine the diversity as well as the structure of the bacterial and fungal communities. Overall, yield, fruit ripeness, and several other agronomical variables were not significantly impacted by the burning treatments. Soil phosphorus was the only parameter with a significant albeit temporary change (1 month after thermal pruning) for soil chemistry. Our results also showed that bacterial and fungal communities did not significantly change between burning treatments. The fungal community was dominated by ericoid mycorrhizal fungi, while the bacterial community was mainly composed of Acidobacteriales, Isosphaerales, Frankiales, and Rhizobiales. However, burning at high intensities temporarily reduced *Septoria* leaf spot disease in the season following thermal pruning. According to our study, thermal pruning has a limited short-term influence on the wild blueberry ecosystem but may have a potential impact on pests (notably *Septoria* infection), which should be explored in future studies to determine the burning frequency necessary to control this disease.

## Introduction

Blueberries are labeled as “functional food,” a group of products that are supposed to have health benefits in addition to the nutrients they provide. In the case of blueberries, studies have shown that their consumption induces positive effects on cognitive, vascular, and gluco-regulatory functions (Krikorian et al., 2010; Whyte et al., 2018; Kalt et al., 2020). Blueberries are among the fruits with the highest content of anthocyanins, pigments from the flavonoids family, which have antioxidant properties (Wu et al., 2006; Kalt et al., 2020). These health benefits may explain why the global demand for blueberries is increasing worldwide (Brazelton, 2013). To satisfy this increase in demand, producers are on constant lookout for agricultural practices that will increase their yield.

Wild blueberries (*Vaccinium angustifolium* Ait. and *V. myrtilloides* Michaux) are cultivated in the east of the United States (Maine) and Canada (Quebec and Maritimes provinces) (Yarborough, 2012), in fields where the plants are native, generally in boreal forests that are cut down or in abandoned farm fields (Yarborough, 2012). Another particularity of this crop is its production cycle as producers have found that pruning wild blueberry after fruit harvesting maintains a higher yield than if the plants were left unmanaged. Following harvest, wild blueberry stems are cut just above the ground level. During the following growing season (the vegetative stage) new stems emerge from the rhizome, and form flower buds, but the plant needs another growing season to produce its fruits. An alternative to mechanical pruning is the use of fire, termed thermal pruning. This method is thought to date back from early settlers who observed Native Americans enhancing blueberry growth by intentionally setting fire to patches of forests containing native shrubs (Chapeskie, 2001; Theriault, 2006). Thermal pruning was adopted by blueberry producers before being gradually replaced by mechanical pruning due to its lower cost (Lambert, 1990). Despite being more expensive, thermal pruning has the capacity of reducing diseases and damage caused by insects (Black, 1963; Blatt et al., 1989), fungal attacks (Blatt et al., 1989; Lambert, 1990; Hildebrand et al., 2016), as well as reducing some weed species (Penney et al., 2008; Smagula et al., 2008; White and Boyd, 2017). This sanitation effect is not observed when using mechanical pruning, which only has a stimulating effect on fruit production (Drummond et al., 2009). Fungal infections, such as monilinia blight (mummy berry), leaf rust, botrytis blight, or *Septoria* and *Valdensinia* leaf spot diseases, can cause severe production losses. Therefore, producers apply fungicides in the infected fields to control and prevent disease propagation (Hildebrand et al., 2016). Thermal pruning could be an alternative to fungicides as it significantly reduces some of these diseases. However, their control can require intense and repeated thermal pruning (Hildebrand et al., 2016). Regarding weeds, prior knowledge of the species present in a field is required to decide whether burning will be effective, as they have varying degrees of resistance. The first category of wild blueberry weeds, which rely on their seed bank to propagate or that have shallow perennating organs, consist of a variety of grasses, such as *Danthonia spicata, Agrostis* spp., *Festuca filiformis*, or moss, such as *Lycopodium* spp., *Poytricum* spp. or *Pleuzorium schreberi*. These weeds are more sensitive to thermal pruning and decrease over time when this practice is employed (Penney et al., 2008). The second, more problematic category consists of weeds that have deep rhizomatous and perennating organs, such as *Comptonia peregrina, Kalmia angustifolia*, or *Cornus canadensis*. These weeds tend not to be negatively impacted by thermal pruning as it is not intense enough to damage their root system (Penney et al., 2008). Some even report an increase of abundance in weeds, such as *Cornus canadensis*, after thermal pruning (Hoefs and Shay, 1981; Penney et al., 2008). Furthermore, White and Boyd have shown that seed viability of three common blueberry weeds (*Tragopogon pratensis, Apocynum androsaemifolium*, and *Panicum capillaire*) is impacted by the temperature and the duration of the treatment, having consistent reduction in germination with temperatures above 200°C (White and Boyd, 2017). Consequently, even when burning can have a positive effect, the intensity of the fire needs to reach a certain threshold in order to witness results.

A detailed investigation on thermal pruning practices demonstrates that there is no consensus on the method to use. First, burning may be done using different kinds of fuels, usually oil (Black, 1963; Smith and Hilton, 1971; Ismail et al., 1981; Ismail and Hanson, 1982; Warman, 1987), propane (Hoefs and Shay, 1981), and/or straw (Black, 1963; Penney et al., 1997). Different fuels have distinct burning temperatures: wheat straw can burn between 200 and 480°C (Wang et al., 2009) when propane can theoretically reach 1,976°C (adiabatic flame temperature of propane in air, Lide, 2005). Second, fuels can be applied in different quantities (ex: 21 t/ha of straw in Penney et al., 1997, vs. 4.5 t/ha in Smith and Hilton, 1971). Third, the season of thermal pruning can vary, usually occurring either in the late fall (Smith and Hilton, 1971; Ismail and Hanson, 1982; Smagula et al., 2008) or early spring (Black, 1963; Hoefs and Shay, 1981; Ismail et al., 1981; Warman, 1987; Penney et al., 1997). All of these parameters have a direct effect on burning intensity. Consequently, without a clear consensus on the method to use, conclusions drawn from the impact of this practice can be contradictory. From a production standpoint, the increase of organic blueberry demand entails a narrower range of synthetic phytosanitary treatments. Thermal pruning for pest management could, therefore, be of particular interests for organic growers. Nevertheless, empirical data are lacking on the fire intensity necessary to observe improvements in blueberry production and to determine if the additional cost implied is worth the investment. Furthermore, depending on its intensity, fire can give rise to significant changes in an ecosystem. Consequently, our study also aimed to verify that thermal pruning did not induce any undesirable changes in the wild blueberry habitat.

Like other members of the *Ericaceae* plant family, wild blueberries grow naturally in acidic soil, with a pH ranging between 4 and 6. These soils are characterized by a high organic matter content, and a low nutrient availability as most of the nutrients required by the plant are bound to organic molecules and are, therefore, not in a readily available source. To prosper in such environments, wild blueberries rely on their root microorganisms for their growth and development, especially on ericoid mycorrhizal fungi (Cairney and Meharg, 2003; Mitchell and Gibson, 2006). Some argue that blueberries are highly dependent on their ericoid symbiotic partnership and could not efficiently grow without it (Cairney and Burke, 1998). Ericoid mycorrhizal fungi produce a panoply of enzymes capable of degrading various organic compounds (Kerley and Read, 1995; Cairney and Burke, 1998; Martino et al., 2018), thereby releasing unavailable nutrients in the soil. These nutrients are then transferred to the plant root, which, in turn, provides photosynthates to the fungi (Pearson and Read, 1973). Bacteria associated with wild blueberries, or *Ericaceae* in general, are much less studied than fungi, but a recent study has shown that some taxa identified in proximity to wild blueberry roots are known nitrogen fixers, which could also be beneficial for blueberries (Morvan et al., 2020). As agricultural practices can modify the soil microbiome, thermal pruning could disturb the wild blueberry beneficial microbial communities. Throughout the literature, the effect of fire on the soil and its microbial communities remains unclear and variable among studies, with most of the research focusing on the effect of forest or prairie fires. Three general effects are usually observed. First, heat superior to 90°C can kill most microorganisms, and fire can, therefore, significantly decrease the biomass of bacteria and fungi (Neary et al., 1999; Hart et al., 2005; Mataix-Solera et al., 2009). Second, if the fire is intense enough to cause significant organic matter combustion, the resulting ash deposit induces an immediate but brief increase in pH and nutrient concentration in soil (Mataix-Solera et al., 2009). These changes will generally favor bacteria over fungi, and bacterial abundance can even increase compared to unburned soil (Mataix-Solera et al., 2009). Finally, intense fires can alter the aboveground plant community, and this turnover will, in turn, impact the microbiomes in relation with these plants, a phenomenon known as plant-soil feedback (Bever, 1994; Dangi et al., 2010; van der Putten et al., 2013). Symbiotic microorganisms could be particularly vulnerable to the loss of their plant host as their fitness depends on the symbiosis. However, the range of changes on microbes caused by fire is highly correlated with the intensity of the fire in question (Neary et al., 1999; Hart et al., 2005; Whitman et al., 2019). Compared to forest or prairie fires, thermal pruning in wild blueberry fields is situated on the low intensity side of the spectrum. Therefore, findings drawn from the numerous previous studies on prescribed fires may not apply to the thermal pruning on wild blueberries. More precisely, the changes observed on soil chemistry and microbial communities should be mitigated by the reduced soil temperature and depth of burn reached during thermal pruning. Burning has been used in other agricultural practices that can be more comparable to wild blueberry thermal pruning, such as post-harvest stubble burning in cotton, sugarcane, rice (Hardison, 1976), as well as wheat and corn (Acree et al., 2020). However, comprehensive studies on the effect of these prescribed fires on soil microbial communities are rare. Acree et al. (2020) found a significant decrease in abundance of soil total bacteria, saprotrophic and arbuscular mycorrhizae fungi 6 h after wheat stubble burning, but a recovery was observed 7 days after the treatment. However, they did not measure any changes in soil microbial abundance for corn stubble burning (Acree et al., 2020). Another study measured the effect of burning in a wheat-lupin-wheat crop sequence on soil biological activities (Alvear et al., 2005). They found a significant increase of urease and dehydrogenase activities in burnt plots and a decrease in acid phosphomonoesterase, and β-glucosidase activities. Microbial carbon and nitrogen were significantly lower in the burnt plots in the 0–200-mm soil layer, 5 months after the burning had occurred (Alvear et al., 2005). These studies, therefore, show that burning in agricultural settings can have effects on the abundance of the microbial communities, as well as on several biological activities. However, each agricultural system is unique, and it is, therefore, difficult to extrapolate on the results obtained in the cited studies.

The aim of our study was to assess the effects of three burning intensities on weed coverage, disease incidence, soil chemistry, and blueberry performance. Furthermore, we characterized the bacterial and fungal communities of the rhizospheric soil to evaluate the repercussions of burning intensity on the microbial community. Our hypotheses were: (1) an increase in pH and nutrients would be correlated with the burning intensity; (2) a reduction of weed coverage and diseases; and (3) that the microbial community would be altered in diversity and/or composition due to the burning intensities. To address these hypotheses, we established an experiment in a random block design in a blueberry research field where three burning intensity treatments and an unburned control were applied at the beginning of spring 2018 during the vegetative stage. Agronomic measurements were taken throughout this year and during the subsequent harvesting year. Soil samples for microbial characterization were collected during the harvesting year, at the end of summer 2019.

## Materials and methods

### Experimental design

The experiment took place at the Research and Teaching Blueberry Field (Bleuetière d'Enseignement et de Recherche) located in Normandin, Quebec (48°49'40.2“N; 72°39'36.9”W) in the North temperate zone (mean temperature: 0.9°C, annual precipitation: 871 mm (Government of Canada, 2021). This blueberry field was established in 2005, and has been attributed for research purposes since 2016. A weather station installed in the field allows to monitor several parameters, such as rainfall and soil temperature. The field has a history of herbicide use; however, no treatments (fertilization, herbicides, pesticides) were applied during the experiment (2018-2019).

The configuration of the experiment followed a randomized complete block design with 16 2.5-m-by-6-m plots organized into four blocks separated by 1-m wide strips. Quadrats of 1 m^2^ were established at the center of the plots, resulting in a distance of 2.5 m between the quadrats (Supplementary Figures 1A,B). Following a mechanical pruning with a blueberry mower (model TB-1072, JR Tardif, Rivière-du-Loup, Canada), the plots were thermally pruned using high-pressure propane burners towed by a tractor on the 15th of May 2018 (Supplementary Figure 1C). The fire prevention authorities (SOPFEU) have very strict policies and allow these fire treatments only during a specific period in order to protect the nearby forests. The home-made liquid propane burner included four individual propane burners that were placed 10 cm above the soil surface and which allowed to burn a 2.5-m length.

Thermal pruning was carried out at three different intensities, with an additional unburned control in each block. The intensity of the burn corresponds to the speed at which the tractor moved over each plot. For the lowest intensity, the speed was set at 1.5 km.h^−1^, while the speed was reduced to 0.5 km.h^−1^ and 0.1 km.h^−1^ for the medium and high intensity, respectively. Considering net heating value of propane of 47 MJ.kg-1 (Linstrom and Mallard, 2001), this fuel consumption represented about 6 580 MJ.ha^−1^ of heat for the lowest intensity (consumption of about 140 kg of propane per hectare at pressure of 15 psi and tractor speed of 1.5 km.h^−1^). At medium and high intensity, this translates to around 19 740 MJ.ha^−1^ and 98 700 MJ.ha^−1^, respectively. Similar to what was found by Vincent and collaborators who used a similar machinery, soil temperatures at 1-cm depth increased by <10°C (Vincent et al., 2018) (Supplementary Figure 1D). One final note to be mentioned is that, in our study, the lowest intensity is already higher than the intensity used by some blueberry producers who drive at 4.8 km.h^−1^ (3 miles.h^−1^), which is more than three times as fast.

### Data acquisition

#### Weeds, disease, and blueberry coverage and blueberry biomass

After the thermal pruning was applied, 1-m^2^ quadrats were set up randomly inside each of the 16 plots. These quadrats were used to implement the point interception sampling method: a quick, low observation bias and a non-destructive method as described in Lévesque et al. (2018). This method is based on the use of a 1-m^2^ stand onto, which a 1-m long aluminum bar, with 10-cm spaced holes, is secured. The operator vertically slides the rod into the holes and records the identity of the plants the rod touches, as well as the number of times the rod impacts a plant before the rod touches the ground. Once the 10 measures are recorded, the bar slides 10 cm farther along the stand, and the processed is repeated over for a total of 100 measurements per quadrat.

This method was used to measure soil, weeds, blueberry coverage over the pruning year (4 measurements), and one measurement at the end of the production year. The coverage is computed using the ratio of the number of intercepts for soil or any given plant over the total number of intercepts. The blueberry biomass was estimated using the blueberry coverage based on the relation found in Lévesque et al. (2018). Blueberry diseases were also measured using this method by recording the rod-intercepted blueberry leaf and stems state.

During the experiment, we identified symptoms corresponding to *Septoria* leaf spot disease and measured its prevalence by computing the ratio of impacted blueberry plant structure intercepts over the total number of blueberry plant structure intercepts.

The weed species identified in the experiment *Cornus canadensis, Maianthemum canadense*, and *Gaultheria procumbens*. Specimens of *Kalmia angustifolia, Apocynum androsaemifolium*, and *Carex sp*. were also observed but not presented in the results as there were too few occurrences for statistical analysis.

#### Soil chemical properties

The organic layer thickness, as well as the soil humidity content, was measured before and after the thermal pruning had occurred. For the organic layer, 10 pseudo-replicates per plot were sampled using a soil corer to a depth of 15 cm, which allowed us to measure organic layer thickness. For soil humidity content, ProCheck moisture sensors were placed between 0- and 4-cm depth, and measures were replicated five times for each plot. Furthermore, soil chemistry was analyzed multiple times during the experiment: 1 and 4 months after burning, as well as an additional sampling during the production year on the 24th of September 2019 to see the effect of burning at a longer scale. Soil samples were extracted using a soil corer at a rate of three pseudo-replicates per plot. The three pseudo-replicates were pooled into one sample once the organic and mineral layers were separated in the lab. Both layers were dried and sieved through a 2-mm mesh. pH was measured using distilled water at a rate of 1:2 (Hendershot et al., 2007). Phosphorus, potassium, calcium, and magnesium were extracted using the Mehlich 3 solution (Ziadi and Tran, 2007). Phosphorus was quantified using colorimetry (Murphy and Riley, 1962), potassium with flame emission spectrophotometry, while an atomic absorption spectrophotometer was used for calcium and magnesium (Perkin Elmer AAnalyst 300, Überlingen, Germany). Finally, carbon and nitrogen concentrations were quantified by dry combustion on a LECO instrument with sieved soil <0.2 mm.

#### Fruit yield

On the 16^th^ of August 2019, we quantified the blueberry yield by harvesting the fruits from within a 50-cm-by-50-cm quadrat in each 16 experimental plots. The number of fruits in 125-ml volume, as well as their ripeness based on visual assessment of fruit color, was also measured for each quadrat.

### Microbial community analyses

#### Sampling

For the microbial community analysis, samples were collected during the harvest year on the 15th and 16th of August 2019. The sampling consisted of two, 10-cm wide, clumps of organic soil per plot, containing blueberry rhizomes. The two pseudo-replicates per plot were handled as separate samples and processed individually onward to evaluate the microbial homogeneity of the plot. The samples were placed into Ziploc bags and kept on ice in a cooler until they were placed at −20°C. Frozen samples were thawed, and the rhizomes were then manually extracted from the clump of soil and placed in separate Ziploc bags. We placed soil aggregates containing roots that were placed into 50-ml Falcon tubes with a pierced cap to freeze-dry the samples. The freeze-dried soil samples were then sieved through 1-mm mesh to remove coarse organic matter and pebbles. The resulting soil material, hereforth named rhizospheric soil, contained thin root fragments, as well as organic matter. The samples were then grounded to homogenize the material to a fine powder (Supplementary Figure 2).

#### DNA extraction and PCR amplification

We weighed, 0.25 g of homogenized rhizospheric soil from each sample for the DNA extraction using the QIAGEN PowerSoil kit (Qiagen, Toronto, ON), following the manufacturer's protocol, with the following modification: for cell lysis, samples were placed in TissueLyser II (Qiagen, Toronto, ON) instead of a vortex for 14 cycles of 45 s each at speed of 4. In addition to the soil samples extractions, we added a negative control/blank sample by replacing the 0.25 g of soil by 250 μL of autoclaved and filtered water. Two mock communities, fungal and bacterial, were also included to be sequenced and act as positive controls. The fungal mock community was designed by Matthew G. Bakker and contains 19 fungal taxa (Bakker, 2018). In our experiment, we used the “even community”, containing an equal amount of the 18-s rRNA gene. The bacterial mock community contained 20 species (Supplementary Table 1), with equimolar counts (10^6^ copies/μL) of 16S rRNA genes (BEI Resources, USA). DNA extracts were stored at −20°C until they could be sent to Genome Québec Innovation Center (Montréal, QC, Canada) for PCR amplification and amplicon sequencing.

For the bacterial community, we targeted the V3-V4 region of 16S rDNA with the primers 341(F) and 805(R), resulting in an expected amplicon size of 464 base-pair long (Mizrahi-Man et al., 2013). Genome Quebec uses four versions of each primer (staggered primers) to increase base diversity in the MiSeq flowcell. For the fungal community, we selected the ITS3KYO(F) and ITS4(R) primers to target the ITS region located between the 5.8S and the LSU region of the ribosomal RNA gene (Toju et al., 2012). The mean amplicon length obtained with this pair of primers was 327.2 base-pair, but this region is known to have varying lengths (Toju et al., 2012). Both sets of primers were coupled to CS1 (forward primers) and CS2 (reverse primers) tags that allow for barcoding. A second PCR was used to add a unique barcode per sample, as well as i5 and i7 Illumina adapters, that bind to the flowcell. Details of the PCR protocols, primers, and adapters sequences, as well as the thermocycler parameters, can be found in Supplementary Methods. Sequencing was conducted on an Illumina MiSeq using a paired-end 2-x-300 base-pair method (Illumina, San Diego, CA, USA).

#### Sequencing data processing

The sequences from the 16S and ITS MiSeq data were downloaded from Genome Québec's platform and checked using the MD5 checksum protocol. We inferred sequence variants from the sequence data using the DADA2 pipeline (Callahan et al., 2016a) in R (R Core Team, 2021). Contrary to the commonly used 97% OTU clustering pipelines, DADA2 provides single-nucleotide resolution of amplicons, which helps to identify chimeras and reduces the rate of false positives (Callahan et al., 2016a). Furthermore, the amplicon sequence variants (ASVs) obtained with DADA2 are not clustered together to a given threshold of similarity and are, therefore, comparable between independent studies, contrary to 97% OTUs as the clustering depend on the dataset analyzed. We processed the mock communities on their own to avoid any influence of our samples on the inferred mock ASVs.

For the fungal dataset, as the ITS region can vary drastically in length, we followed the DADA2 ITS Pipeline workflow. The first step of the pipeline consists in using cutadapt (Martin, 2011) in order to remove the primers and their reverse complements by indicating the primers' nucleotide sequences. Once the primers removed with cutadapt, we proceeded with the filtering step using maxEE(2,2) and minLen(50), which removes sequences that are shorter than 50-nucleotides long. Regarding the bacterial dataset, as staggered primers were used, we followed a similar pipeline as the one used for the fungal dataset relying on cutadapt to remove the different versions of the primers. For the filtering step, we set truncLen to (270, 240) and maxEE (2, 2) based on a visual assessment of the quality profiles. After the different filtering steps, both bacterial and fungal datasets followed the same pipeline that used the default settings except those mentioned hereafter. In the learning error rates step, we used randomize = TRUE; in the sample inference step, we used “pseudo” as a pooling method and, accordingly, the “pooled” method for the bimera removal step. To add a taxonomy assignment to our inferred ASVs, we used the implemented naïve Bayesian RDP classifier with the *assignTaxonomy()* function. For the bacterial dataset, we used the SILVA reference database and two UNITE databases for the ITS sequences. Based on the taxonomy obtained, we removed non-bacterial ASVs, as well as ASVs that were annotated as chloroplast and mitochondria in the 16S dataset. The UNITE fungal reference database resulted in every ASVs to be labeled as Fungi, but many sequences did not have an assigned Phylum. We compared this taxonomic assignment to a second one obtained by using the UNITE's eukaryotic reference database. Most of the unknown phyla fungi were labeled as non-fungal when using the eukaryotic reference database and were, therefore, removed in the ITS dataset. We then proceeded to further refine our datasets by removing singletons and doubletons (ASVs with a total abundance of 1 or 2) as they may be sequencing artifacts. Details of sequence data processing and mock community analyses can be found in the Supplementary Information.

### Statistical analyses

All analyses were performed in R version 4.1.1 (R Core Team, 2021), and figures were generated using the ggPlot2 R package (Wickham, 2016).

#### Soil and agricultural data

To test the effect of burning intensity on soil and agricultural data, we used either linear mixed models or generalized linear mixed-effects models. For data with a unique sampling date (blueberry performance apart from biomass), we computed linear mixed models (LMM), with blocks as random effects, followed by ANOVA tests to detect statistical differences between burning intensities. We used the lmerTest R package (Kuznetsova et al., 2017), which implements a Type III ANOVA using the Satterthwaite's method. For data with multiple sampling dates (weeds, disease, or soil chemistry), we either used LMM or generalized linear mixed-effects models (GLMM) using the *glmer()* function of lme4 R package (Bates et al., 2015). The choice of the model depended on data distribution, and appropriate transformations (arcsin, log or square-root for LMM) or regression (Poisson for GLMM) were employed. Burning intensity, date, and the interaction between the two were set to be the fixed effects, and both experimental blocks and plots were set as random effects, with the plots nested in the experimental blocks. For the GLMM, we used the *ANOVA()* function of the car R package (Fox and Weisberg, 2019) to perform a Type 3 Wald chi-square test. When the tests showed a moderate evidence of an effect (*P* < 0.05) of burning intensity or the interaction of burning intensity on the variable of interest, we reiterated the models for each individual date and removed the date for the fixed factors and the plot from the random factors and checked for significant differences between treatments using the *post hoc* Tukey tests.

#### Microbial data

To check if our sequencing depth captured most of the bacterial and fungal communities, we generated rarefaction curves using the *rarecurve()* function of the vegan R package (Oksanen et al., 2020) (Supplementary Figure 3). We used phyloseq (McMurdie and Holmes, 2013) to facilitate data handling and to generate alpha diversity and beta diversity plots. We used Metacoder (Foster et al., 2017) for a more thorough analysis of the taxonomy of each dataset by plotting the ASV number and the relative abundance per taxa up to the genus level using the *heat_tree()* function.

#### Phylogenetic trees

To take into account the phylogenetic distance between the inferred ASVs and use Unifrac distances, we built phylogenetic trees following the method used in Callahan et al. (2016b). First, a sequence alignment was generated using *AlignSeqs()* from DECIPHER (Wright, 2016). We then used the phangorn package (Schliep, 2011) to compute a distance matrix under a JC69 substitution model using *dist.ml()*. A neighbor-joining tree was then assembled onto which we fitted a generalized time reversible with the Gamma rate variation (GTR + G + I) model using *optim.pml()*. The resulting trees were then added to their respective phyloseq objects.

#### Pseudo-replicate similarity and beta diversity

To compare the microbial communities in the pseudo-replicates (two samples originating from the same plot), we relied on a beta diversity analysis using four different dissimilarity metrics. First, to take into account the compositional aspect of sequencing data, we used the Aitchison distance (Quinn et al., 2018). Second, we used the Hellinger distance as its traditionally used in ecology (Legendre and Gallagher, 2001), and, finally, we used the unweighted and weighted Unifrac distance to account for the phylogenetic resemblance of the communities. Ordinations allowed to visualize the pseudo-replicates community resemblance using principal components or principal coordinates analyses (PCA or PCoA). The pseudo-replicates were merged together after this analysis by summing their sequence abundance using *merge_samples()* from phyloseq. We then proceeded to the same analyses to estimate the similarity between the microbial communities originating from different burning treatments. To test the significance of the differences observed, we used vegan's *adonis2()* function with 999 permutations, taking into account the experimental blocks. The homogeneity of dispersion assumption was checked using *betadisper()* and *permutest()* both in the vegan R package (Oksanen et al., 2020).

#### Alpha diversity

The Simpson and Shannon-Weaver alpha diversity indices were computed using the *plot_richness()* function of phyloseq. Both alpha diversity analyses were determined with and without rarefaction using *rarefy_even_depth()* as sequence abundance can impact alpha diversity measures. Statistical differences between different burning intensities were checked using one-way ANOVA's in a similar fashion than for soil and agricultural data (see above).

#### Core microbiota

To detect the shared and unique ASVs of each thermal treatment, we used *ps_venn()* from the MicEco R package (Russel, 2021). It allowed us to plot both the ASV number and the relative abundance that they represent in the datasets.

#### Representative taxa

Metacoder was used as a finer approach than the beta diversity analysis to detect significant differences in each taxonomic rank between the burning intensities (Foster et al., 2017). The *compare_groups()* function was used after quantifying the per-taxon relative abundance using *calc_taxon_abund()*. This function computes a non-parametric Wilcoxon Rank Sum test to detect differences in taxon abundance in different treatments. In order to take into account the multiple comparisons, we corrected the *P*-values of the Wilcoxon Rank Sum test with false discovery rate (FDR) correction using the *p.adjust*() function of the stats R package (R Core Team, 2021). After setting a threshold of 0.05 for the *P*-value, we used the *heat_tree_matrix()* to plot statistically different taxa base on their abundance using the log2 ratio of median proportions.

Additionally, we used DESeq2 to identify differentially abundant ASVs between two groups of samples (Love et al., 2014). In our case, we used the entire datasets for the analysis but chose to only present the comparison of the negative control to the highest burning intensity as the difference in treatment between the two conditions is maximized and so is the potential effect of burning. We agglomerated the ASVs at the species level prior to the analysis (see indicative species analysis for more details). As sequencing data are known to be sparse, some ASVs can be absent in one of the conditions. DESeq2 calculates a log 2-fold ratio by considering that the absence of the ASV is due to non-detection and spikes the data to allow the computation of the log 2-fold ratio. However, it is impossible to know with certainty if the absence is biologically supported (in which case, the log 2-fold ratio has no meaning) or if it us due to a lack of detection. Therefore, in the case where an ASV was absent from one condition, the computed log 2-fold ratio value was changed to “– INF” or “+ INF,” depending on which condition the ASV was absent from. To increase our confidence in the taxa found in this analysis, we present only the significant taxa that were present in at least three of the four replicates of a condition. We also proceeded with an indicative species analysis, which is presented in the Supplementary Information.

### Accession numbers

Raw sequences have been deposited in the GenBank SRA database under the accession No. PRJNA803472.

## Results

### Effects of burning intensities on blueberry's agronomic variables

#### Blueberry performance

**Figure 1:**
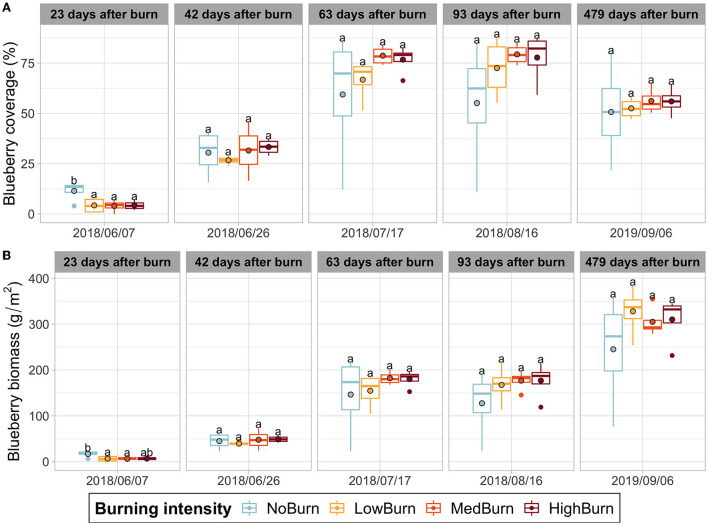
Blueberry coverage and biomass production over time. Mean value is indicated with a black-circled dot. **(A)** Wild blueberry coverage. **(B)** Wild blueberry biomass production computed using the relation found by Lévesque et al. (2018). Significant difference in each sampling date is indicated by letters according to *post hoc* Tukey tests.

Burning delays the vegetative recovery in spring with a significantly reduced blueberry coverage for the three burning treatments compared to the control (Figure 1A). As blueberry biomass is derived from the blueberry coverage determined using the point intercept method, we also observed a similar significant difference in blueberry biomass between the burning treatments and the control (Figure 1B). However, this difference subsided quickly in the next acquisition date, and both blueberry coverage and biomass of the four treatments remained homogenous throughout the rest of the experiment (Figure 1).

The high disparity between the replicates, especially in the unburned control, did not allow us to establish an effect of burning on shoot density (*P* = 0.2619), which varied between 322 per 1 m^2^ for the control and 450. stems per 1 m^2^ for the medium intensity treatment. Similarly, for shoot growth, a high variability in replicates of the unburned control prevented to find a significant effect of burning on shoot growth (*P* = 0.6412), although it tended to increase with burning with a mean length of 20.7 cm for unburned controls compared to 23.2, 23.5, and 23.7 cm at low, medium, and high burning intensities, respectively.

The range of blueberry yields observed in a given treatment was too disparate to observe an effect of burning intensity (*P* = 0.9516). Blueberry yields were higher for the control than for the different burn treatments with 2.750 T.ha^−1^ vs. 2.100, 2.275, and 2.325 T.ha^−1^ in order of increasing burning intensity.

Burning intensities did not increase the proportion of ripe fruits, as determined by the ANOVA test (*P* = 0.2112). Unburned control had 77.9% of the ripe fruits compared to 63.8% for the low-intensity burn treatment, while 55.5% and 55.9% of the fruits were ripe in the medium and high-intensity treatments, respectively. Burning had no effect on the average weight of fresh ripe fruits (*P* = 0.1704), which was similar for the unburned control (0.283 g/125 ml) and medium- and high-intensity treatments (0.288 g/125 ml for both) but was lower for low-intensity burn (0.240 g/125 ml).

#### Weeds and disease

**Figure 2:**
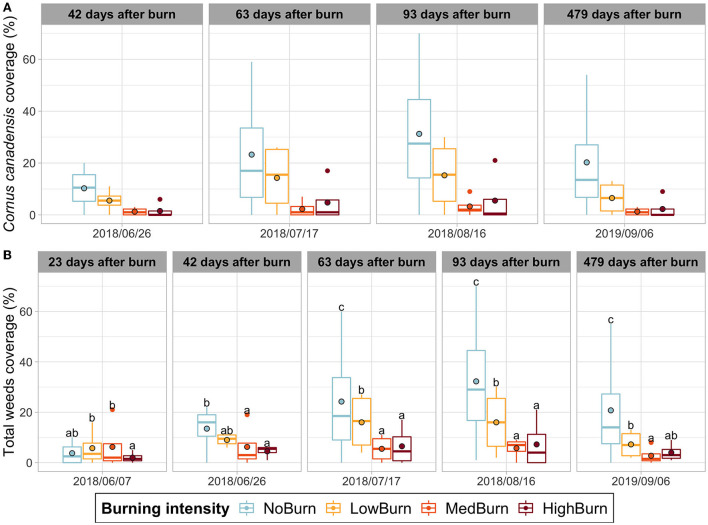
Weed coverage over time. Mean value is indicated with a black-circled dot. **(A)** *Cornus canadensis* coverage. **(B)** Total weed coverage (pooled coverage of all the weed species observations). Significant difference in each sampling date is indicated by letters according to *post hoc* Tukey tests.

*Cornus canadensis* was, by far, the most common and dominant weed species recorded, with a peak abundance in August 2018 (3 months after burning) when its coverage reached an average of 31.2% of the plots surface in the unburned controls. Although burning tends to decrease *C. canadensis* presence, the results were not statistically significant due to high variabilities (*P* = 0.3499). In low-intensity treatment, *C. canadensis* coverage was 15.2%, and 3.1% in medium, and 5.7% in high-intensity treatments. The date had a significant effect (*P* = 1.226e-08), but the interaction between burning and the date was not significant (*P* = 0.5907) (Figure 2A). Regarding the other two weed species found, burning did not have an effect on *Maianthemum canadense* (*P* = 0.8208) nor on *Gaultheria procumbens* (*P* = 0.9730) (Data not shown).

We did not test the effect of burning on the other weed species found as they were too sparse in our sampling. Instead, we summed the coverage of all the weeds species recorded to test the effect of burning on total weed coverage. The ANOVA (Type 3 Wald chi-square) test showed strong evidence of an interaction between the sampling date and burning intensity (*P* = 1.031e-06). The *post hoc* Tukey tests identified statistical differences between the unburned control and the medium and high burning intensities for four out of the five sampling dates. The low-burning intensity also significantly reduced the total weed coverage compared to the unburned control in the last 3 sampling dates but to a lesser extent than the more intense burning intensities (Figure 2B).

**Figure 3:**
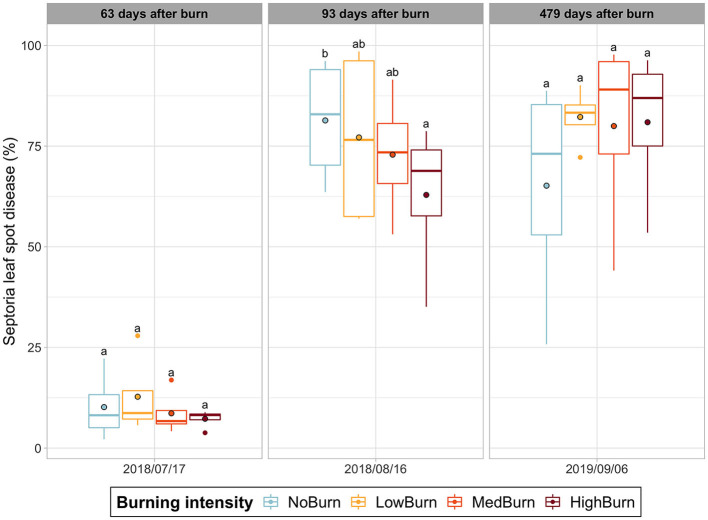
Septoria leaf spot disease incidence over time. Mean value is indicated with a black-circled dot. Significant difference in each sampling date is indicated by letters according to *post hoc* Tukey tests.

The *Septoria* leaf spot was the only disease detected with recognized symptoms. We found a significant decrease (*P* = 0.0204) of around 23% of *Septoria* leaf spot disease between the unburned control (mean coverage of 81.4%) and the highest intensity burn treatment (mean coverage of 62.9%), 3 months after the burning treatment had occurred (Figure 3). This difference did was not maintained as we did not find any significant difference between treatments at the subsequent date of sampling.

#### Soil chemistry

**Figure 4:**
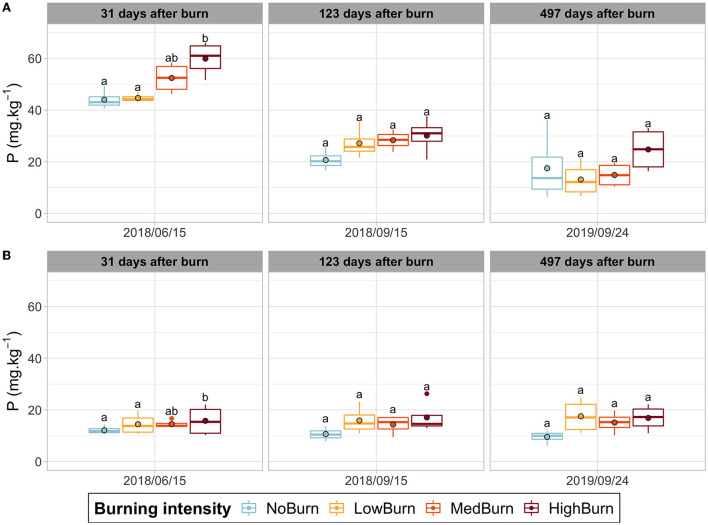
Phosphorus concentration in soil over time. Significant difference in each sampling date is indicated by letters according to *post hoc* Tukey tests. Mean value is indicated a black-circled dot. **(A)** Phosphorus concentration in the organic layer. **(B)** Phosphorus concentration in the mineral layer.

Burning had no significant effect on either organic layer thickness or its humidity content (Supplementary Figure 4). Similarly, we did not find any significant effect of burning on soil pH, which ranged from 3.9 to 4.8 in the organic layer and from 4.6 to 5.1 in the mineral layer, depending on the date of sampling (Supplementary Figure 5). The total carbon did not vary significantly due to burning neither in the organic nor in the mineral soil layer. As expected, the organic layer contained a higher carbon fraction, ranging from 10 to 25% in the organic layer and from 0.85% to 1.1% in the mineral layer (Supplementary Figure 6). Regarding the total soil nitrogen, we did not observe any effect of burning for both soil layers. In the organic layer, the nitrogen content was highest in June 2018 and lowest in September 2018 (0.83% and 0.32%), while, in the mineral layer, it peaked at 0.048% in September 2018 and was the lowest in September 2019, with an average of 0.02% (Supplementary Figure 7). For phosphorus (P) soil content in the organic layer (Figure 4A), we observed that burning significantly influenced P content between the highest burning intensity (59.9 mg.kg^−1^) and the lowest intensity and unburned treatments (44.7 mg.kg^−1^ and 44. mg.kg^−1^, respectively), in June 2018 (*P* = 0.001785). A general trend can be seen as an increased intensity of burning tends to correlate with a higher P content in the organic layer. In the mineral layer, however, we did not observe any change in P content (ranging from 9.6 to 17.7 mg.kg^−1^) due to burning intensity (Figure 4B). For potassium, magnesium, and calcium, we obtained a significant difference between the low-intensity treatment and the control for the last sampling date (Supplementary Figures 8–10). For magnesium and calcium, we also obtained a significant difference in the mineral layer for this date (Supplementary Figures 9, 10). However, we do not observe any significant differences for the two other dates nor for the more intense thermal intensities. In the organic layer, potassium ranges from 145 mg.kg^−1^ to 394 mg.kg^−1^, depending on the sampling date, while ranging from 4.8 mg.kg^−1^ to 24.1 mg.kg^−1^ in the mineral layer (Supplementary Figure 8). Magnesium concentrations were found to be lower in September 2019 and highest in June 2018, with 109 mg.kg^−1^ and 319.5 mg.kg^−1^ in the organic layer and 3.5 mg.kg^−1^ and 7.7 mg.kg^−1^ in the mineral layer (Supplementary Figure 9). Finally, calcium ranged from 820.7 mg.kg^−1^ to 2525.3 mg.kg^−1^ in the organic layer and from 10.1 mg.kg^−1^ to 48.2 mg.kg^−1^ in the mineral layer (Supplementary Figure 10).

### Microbial communities

#### Taxonomic diversity of fungal and bacterial communities

**Figure 5:**
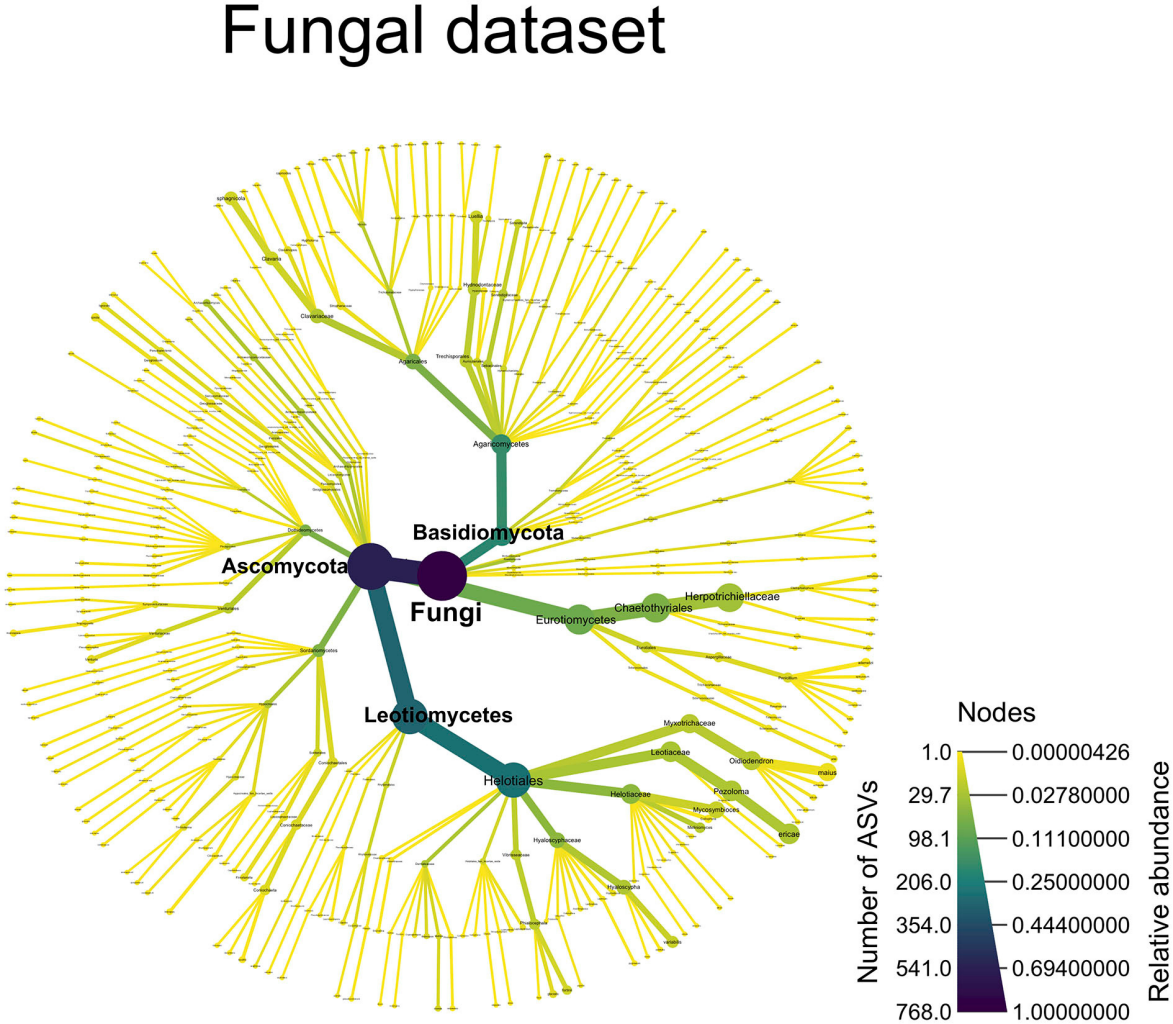
A taxonomy overview of the fungal community. This figure displays all of the fungal ASVs, which were assigned to a taxonomic level [ASVs not assigned at a particular taxonomic level (NAs) are not shown]. The color of the edges and nodes indicates the number of ASVs found at a given taxonomic level, with darker color indicating more ASVs and lighter colors indicating fewer ASVs. The size of the edges and nodes indicates the relative abundance of the ASVs assigned to a particular taxonomic level with wider edges/nodes, indicating a high relative abundance and narrower edges/nodes, indicating a low relative abundance.

From the ITS dataset, Figure 5 shows a dominance of Ascomycota (P)—Helotiales (O), both in terms of relative abundance (46.8%) and ASV numbers (244). Two Helotiales species particularly stand out: *Pezoloma ericae* and *Oidiodendron maius*, represented by 25 and 15 ASVs, respectively, and totaling 12.8% and 9% in terms of relative abundance (RA). Chaetothyriales is the second predominant order belonging to Ascomycota; most of its ASVs belonged to the Herpotrichiellaceae family (32 ASVs, 27% RA). In the Basidiomycota, the Agaricales order stands out, with 61 ASVs but only 5.7% RA; *Clavaria sphagnicola* represented most of this relative abundance (3.8% RA dispatched into 9 ASVs).

**Figure 6:**
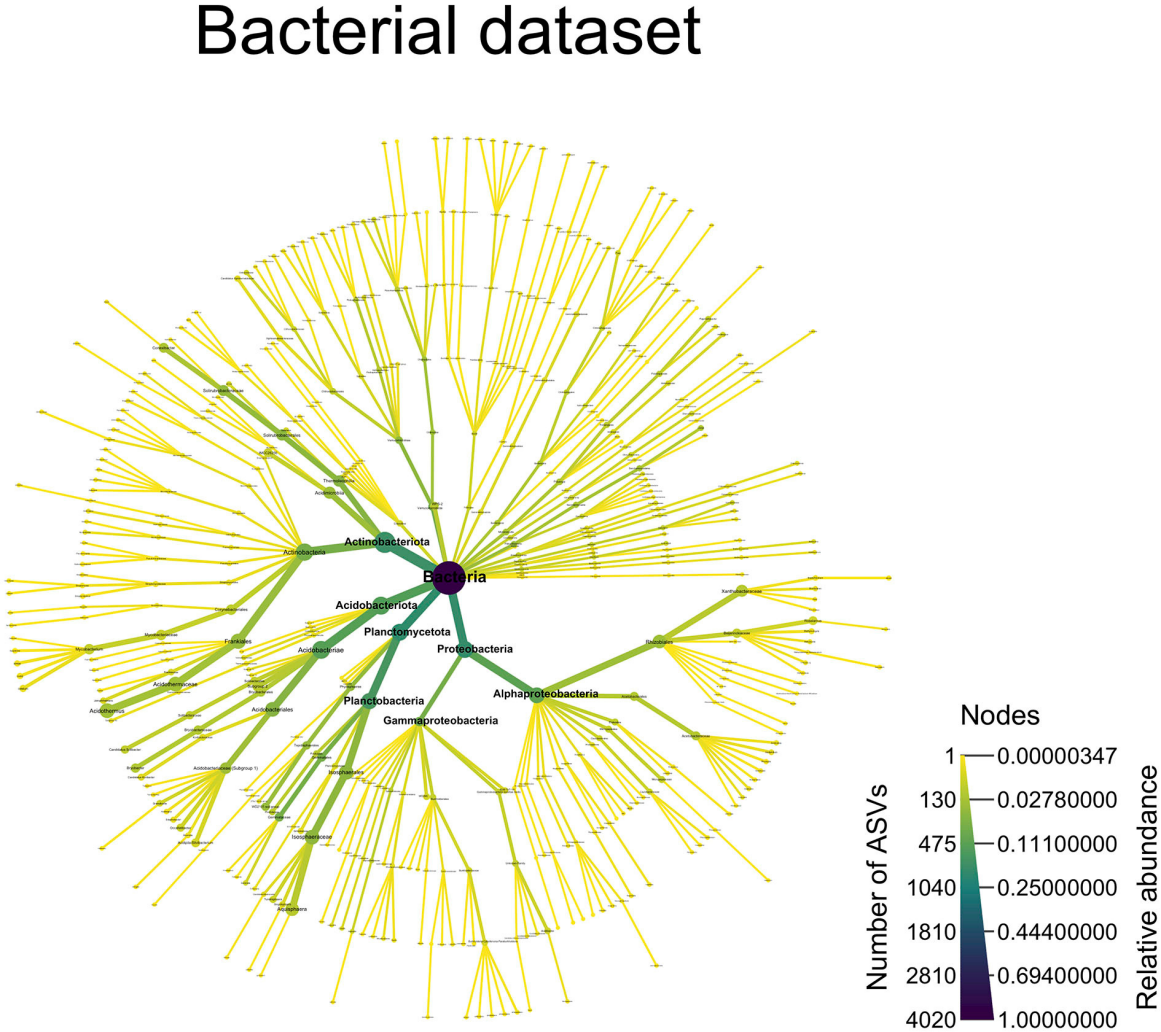
A taxonomy overview of the bacterial community. This figure displays all of the bacterial ASVs, which were assigned to a taxonomic level [ASVs not assigned at a particular taxonomic level (NAs) are not shown]. The color of the edges and nodes indicates the number of ASVs found at a given taxonomic level, with darker color indicating more ASVs and lighter colors indicating fewer ASVs. The size of the edges and nodes indicates the relative abundance of the ASVs assigned to a particular taxonomic level, with wider edges/nodes indicating a high relative abundance and narrower edges/nodes indicating a low relative abundance.

For the bacterial dataset, the predominant taxa were Actinobacteriota (33% RA, 705 ASVs), Acidobacteriota (22% RA, 518 ASVs), Pseudomonadota (formally Proteobacteria) (18.9% RA, 768 ASVs), and Planctomycetota (18.4% RA, 798 ASVs) (Figure 6). The Frankiales order was preponderant in the Actinobacteriota, with 14% RA and 175 ASVs mainly assigned to the *Acidothermus* genus (12.9% RA, 134 ASVs). The Acidobacteriota was dominated by Acidobacteriales order (12.5% RA, 236 ASVs). In the Pseudomonadota phylum, the Rhizobiales order (10.4% RA, 150 ASVs) was preponderant, which, in turn, was dominated by the *Roseiarcus* genus (2.5% RA, 31 ASVs). Finally, for Planctomycetota, the Isosphaerales order dominated by RA but not in terms of ASV richness (12.4% RA, 209 ASVs), as the Gemmatales order contained 304 ASVs but 2.7% in RA. The most abundant Isosphaerales belonged to the *Aquispharea* genus (10.1% RA, 81 ASVs).

#### Bacterial and fungal core microbiomes

Less than a third of the fungal ASVs (28.9%) are shared by all burning treatments, but they represent most of the data, with 81% RA. Each treatment had unique ASVs, ranging from 69 for the low-intensity burn to 144 for the unburned treatment; however, taken together, these unique ASVs represented a low RA in the fungal community (Supplementary Figure 11A). Among bacteria, 44.6% of the ASVs were found in all of the treatments and represented 95% of the RA. The unique ASVs in each treatment ranged from 294 to 522 but had a low abundance as their sum did not exceed 1% in terms of relative abundance (Supplementary Figure 11B).

#### Pseudoreplicate similarity

The resulting ordinations based on different dissimilarity metrics show a certain heterogeneity in both the fungal and bacterial communities, as pseudo-replicates originating from the same plot do not group close together in the ordination (Supplementary Figures 12A,B).

### Effect of burning intensity on the microbial communities

#### Alpha diversity

There was no evidence that burning affected the fungal community (Shannon, *P* = 0.473, Simpson, *P* = 0.698). The assumptions were satisfied for all ANOVA's, except for the heterogeneity of variances in the Simpson's measures. For both diversity metrics, the dispersion in each treatment was quite large, especially for the low-burning intensity treatment, where Plot 7 had a lower alpha diversity than the rest of the plots belonging to this treatment (Supplementary Figure 13A). Similarly, no significant effect of burning intensity was found for the bacterial community diversity (Shannon, *P* = 0.194, Simpson, *P* = 0.196), with a tendency for a higher alpha diversity for the unburned control (Supplementary Figure 13B). Overall, the fungal alpha diversity indices were lower than the bacterial indices. Furthermore, rarefying the sequence abundance to an even depth did not change the tendencies observed nor the outcome of the statistical tests (data not shown).

#### Beta diversity

**Figure 7:**
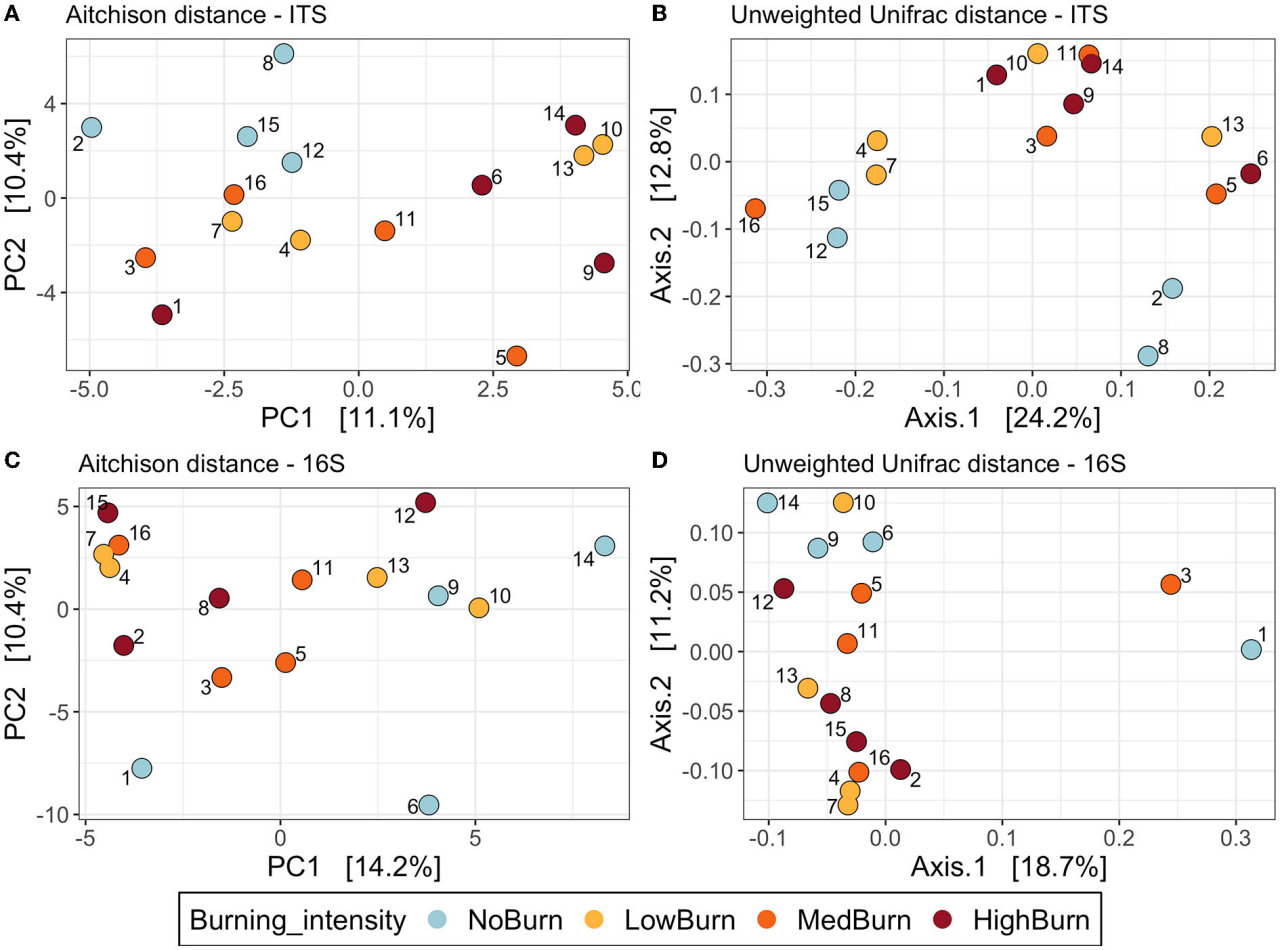
Beta diversity ordinations of the fungal and bacterial communities. **(A,B)** represent principal component analysis (PCA) and principal coordinate analysis (PCoA) ordinations of the fungal community using the Aitchison distance (Euclidean distance on centered-log ration transformed abundances and the unweighted Unifrac distance based on phylogeny dissimilarity, respectively. **(C,D)** represent the same ordinations but of the bacterial community.

We used four different metrics (Aitchison, Hellinger, unweighted and weighted Unifrac) to visualize and test the effect of burning intensities on microbial community compositions using ordinations and PERMANOVAs. For the ITS dataset, the visualization of the Aitchison PCA and the unweighted Unifrac PCoA showed a separation of the unburned plots and the intensely burned plots (Figures 7A,B). However, regardless of the distance used, we found no evidence of the effect of burning intensity in shaping the fungal community composition when using PERMANOVA tests (*P* > 0.05). Finally, for each of the four distance metrics used, the variance in terms of community composition explained by burning intensity was around 20%. For the bacterial community, we also observed a separation of the unburned plots and the high-intensity burned plots in the Aitchison PCA, unweighted Unifrac PCoA (Figures 7C,D), as well as in the Hellinger PCA (data not shown). However, as for the fungal community, we did not find any statistical evidence of the effect of burning on shaping the bacterial community (*P* > 0.05). The variance explained by burning intensity was also around 20% for the four metrics computed for the bacterial dataset.

#### Representative ASVs

Although the beta diversity and PERMANOVAs indicated no significant differences in either the fungal or bacterial communities between each burning treatment, we looked at a finer level in order to detect ASVs that could be representative of a specific burn treatment. First, the differential heat tree plot showed no significant difference in the log 2-fold ratio of ASVs regrouped based on their taxonomy (FDR *P* > 0.05). The indicative species analysis using ASVs agglomerated at the species level found no evidence for indicative ASVs (FDR *P* > 0.05) whether without or with relevant site combinations. Finally, the DESeq 2 analysis on the species level agglomerated ASVs (used to detect differential abundance between the highest burning intensity and the negative control) identified, with moderate evidence, six fungal ASVs but no bacterial ASVs impacted by burning intensity (FDR *P* < 0.05). Half of these fungal ASVs were absent from the other condition, the other three being present in both treatments: two taxa belonging to the *Coniochaeta* genus were more abundant in the highest burning treatment, and one *Pseudoanungitea sp*. was more abundant in the negative control treatment (Supplementary Figure 14).

## Discussion

### Thermal pruning had no significant impact on blueberry performance or agronomic variables, even at the highest intensities

Thermal pruning had a negative effect on wild blueberry spring vegetative recovery with significantly lower coverage and biomass for the three burning intensities compared to the unburned control. However, the difference disappeared over time, and all treatments had similar coverage and biomass during the rest of the experiment. Thermal pruning did not significantly impact stem density and growth, although we did see a slight increase in both variables, with increasing burning intensity. Additionally, fruit yield and ripeness were not significantly different when comparing treatments. Finally, we did not observe any trend in the weight of ripe fruits. Overall, these results indicate that wild blueberry recovers from thermal pruning very well. This was expected as wild blueberries populations are known to thrive after forest fires (Chapeskie, 2001; Wood, 2004). Furthermore, studies have shown that the use of thermal pruning in wild blueberry agricultural settings has promoted yields (Smith and Hilton, 1971; Warman, 1987; Penney et al., 1997). In our study, we do not measure any significant difference in blueberry performance during the harvesting year (yield, ripe fruit weight, blueberry shrub biomass) when we compared the three treatments (thermal pruning after mechanical pruning) to the control, which was only mechanically pruned. Therefore, burning did not appear to contribute to a direct promotion of blueberry performance that mechanical pruning did not already provide. In fact, Smith and Hilton argued that the increased yield observed in their study, comparing thermal pruning to mechanical pruning, originated from the nutrients released from straw ash deposition (Smith and Hilton, 1971).

### Thermal pruning has a temporary phytosanitation effect on *Septoria* leaf spot disease, but this effect is unclear on weeds

Due to the high discrepancy in the control replicates, which likely leads to a lack of statistical difference with the other burning treatments, the reduction of *Cornus canadensis* incidence observed must be taken with caution. The other two recorded weed species (*Maianthemum canadense* and *Gaultheria procumbens*) do not seem to have been affected by thermal pruning. When all weed species were pooled together, we did observe a significant difference between the unburned control and the burning treatments. However, the total weed coverage trend closely followed the *C. canadensis* trend as it was the predominant weed species in our plots. Although the experiment followed a random block design, there is a chance that the weed population density was disparate between the treatments, as we have no data on the preexisting *C. canadensis* population in our plots. Additionally, *C. canadensis, M. canadense*, and *G. procumbens*, like wild blueberries, are rhizomatous plants that primarily spread *via* their rhizome growth rather than by seed germination (Lee, 2004; Moola and Vasseur, 2009). As the highest intensity burn only increased the temperature by 4°C at a depth of 1 cm (Supplementary Figure 1D), the majority of the rhizomes should have been left intact. Thus, we would expect these weed species to be largely resistant to our thermal pruning treatments, depending on how deep their rhizomes are (Flinn and Wein, 1977; Penney et al., 2008). Finally, our results are in disagreement with previous studies, which report either no impact or a positive effect of thermal pruning on *C. canadensis* (Hoefs and Shay, 1981; Penney et al., 2008). Therefore, we suggest thermal pruning would not explain why we observed a decrease in weed coverage due to burning intensity. Accordingly, we cannot draw conclusions on the effect of burning on the weed species we observed in our experiment, and this non-significant reduction in *C. canadensis* coverage should be investigated in-depth for validation. *C. canadensis* can be highly competitive in wild blueberry farms (Hall and Sibley, 1976; Yarborough and Bhowmik, 1993); therefore, if thermal pruning does reduce its population, this practice offers a great solution for organic producers, who are not allowed to use herbicides.

*Septoria* leaf spot disease (*Septoria* LSD) causes a premature leaf drop, which can result in a loss of crop yield. We only observe a decrease of its incidence (negatively correlated with burning intensity) during the vegetative summer (3 months after the thermal pruning), with a significant difference between our negative control and the high-intensity burn. However, this significant change in occurrence did not translate to reduced yield for our negative control. *Septoria* sp. propagates through spores produced on pycnidia that develop on pre-infected dead leaves from the soil litter (Hildebrand et al., 2016). We can hypothesize that burning diminished the number of viable spores, thus reducing the disease propagation (Hardison, 1976; Hildebrand et al., 2016). However, we still witnessed a high coverage of this disease with a mean incidence of 62.9% even for the high-intensity burn. Furthermore, there was no significant difference during the harvesting year where the burning treatments actually had higher *Septoria* LSD coverage than the negative control. This can be explained by the fact that plots were located close to each other, and there were no means to prevent the spores arising from the more infested unburned control to contaminate the less-infected high-intensity burned plots. Interestingly, we did not capture any *Septoria sp*. in the fungal rhizosphere community even though Septoria LSD symptoms were detected in our plots. As plant litter was excluded from our soil sampling for DNA sequencing, this could explain why we did not capture any *Septoria* sp. DNA sequence.

### Thermal pruning increased soil phosphorus content but did not significantly influence other elements

Overall, soil chemistry was not impacted by the burning treatments regardless of the burning intensity used. Apart from the phosphorus content, which increased significantly with burning intensity in the organic layer 1 month after the treatment, there was no clear effect of thermal pruning on the rest of the elements measured (C, N, K, Mg, and Ca) nor on soil pH. Previous studies on wild blueberry thermal pruning have shown that there was an increase in pH, phosphorus, and potassium, following the burning treatment when straw was added (Smith and Hilton, 1971). An increase in calcium and phosphorus in the organic soil layer was also observed in an experiment using a propane burner (Hoefs and Shay, 1981). However, these changes in nutrient content resulted from the combustion of organic matter, which was very limited in our plots. For instance, contrary to Smith and Hilton, we did not witness any ash deposit after the burning treatments, suggesting that combustion in our experiment was low (Smith and Hilton, 1971).

### Rhizosphere bacterial and fungal communities were homogenous throughout the thermal pruning treatments, more than a year after the burning treatment

The alpha diversity analyses showed no significant difference between the burning intensities treatments either for bacterial or fungal communities. The alpha diversity indices were higher for bacteria than for fungi, with a mean range of 6.35–6.5 vs. 3.1–3.7 for the Shannon-Weaver Index and 0.996–0.997 vs. 0.89–0.95 for the Simpson reciprocal index. This difference is common, as alpha diversity indices rely on richness and evenness and that bacteria communities are commonly richer than fungi in soil, even though a low soil pH tends to generally decrease bacterial diversity (Fierer and Jackson, 2006; Rousk et al., 2010).

We obtained a similar outcome with our beta diversity analyses, regardless of the distance metric used. Furthermore, the DESeq2 analysis, which aimed at detecting ASVs that were differentially abundant between the negative control and the high-intensity burning treatment, only identified six fungal taxa, two of which were exclusive to the control (*Mycena sp*. and an unknown genus) and one of which was exclusive to the high-intensity burning treatment (*Lachnum* sp.). The other three ASVs represented a very small portion of the relative abundance (RA), with 0.14% RA for *Pseudoanungitea* sp, 0.25% RA for *Coniochaeta boothii*, and 1.35% RA for *Coniochaeta* sp. Therefore, their differential abundance between the negative and most extreme treatment does not induce a significant shift in community diversity. The two *Coniochaeta* sp. ASVs were more abundant in the high-intensity burn, which could be explained by the fact that this genus contains “fire-induced” species (Wicklow, 1975). The *Pseudoanungitea* genus (more abundant in the control) was described in 2018, and one species (*P. vacinii*) was isolated from the stem of *Vaccinium myrtillus*, a closely related plant from wild blueberry (Crous et al., 2018). Most of *Pseudoanungitea* are saprotrophic (Shen et al., 2020), but a sensitivity to fire has not been documented.

A shortfall of our study is that we sequenced the microbial communities more than a year after (~15 months) the burning treatments were performed, which precluded us from detecting an immediate shift in microbial communities. Wild blueberry generally grows in soil with low-nutrient availability, and the plants must rely on their microbial communities, especially on ericoid mycorrhizal fungi, to uptake sufficient amounts of nutrients (Cairney and Meharg, 2003). Although wild blueberry rhizomes act as a nutrient source during growth, Grelet and collaborators showed that *Vaccinium* sp. do need exogenous nitrogen during the harvesting year, and cannot solely rely on its nutrient reserve (Grelet et al., 2001). Therefore, we chose to sequence the communities present at the end of the production cycle, at the time of harvest. Ideally, we would have sequenced before the burning treatment and throughout the two growing seasons to have a thorough analysis of the impact of thermal pruning on bacterial and fungal communities. Our study cannot confirm that thermal pruning used at these intensities did not impact these fungal and bacterial communities, as there is a possibility that the communities are resilient and recovered from the initial disturbance caused by thermal pruning. Moreover, we believe that, if there was an initial disturbance, it would have been very mild, such as observed for soil phosphorus content. Our results show that there was a very limited impact of burning on soil pH and nutrient content. In addition, the highest fire intensity was not sufficient enough to cause a reduction in the organic layer depth or its humidity. Studies observing shifts in microbial communities after fires also see changes in soil chemistry, humidity, and temperature, as well as a shift in the plant community (Hart et al., 2005; Dooley and Treseder, 2011; Dove and Hart, 2017; Whitman et al., 2019). However, in our study, thermal pruning is not inducing plant succession comparable to intense forest fires. Finally, the temperature at 1-cm depth increased only by 4°C with the highest intensity (Supplementary Figure 1D), while increasing as high as 80°C at the surface (data not shown). Although a good proportion of microbes at the surface may have been killed, the temperature in the soil did not rise high enough to cause a high mortality in the rhizosphere microbiome (Neary et al., 1999). Consequently, we can either hypothesize that thermal pruning in our study did not impact fungal and bacterial rhizosphere communities of wild blueberries or, if it did, we can affirm that both communities are resilient 15 months after burning.

Despite the fact that some plots were devoid of *C. canadensis*, while others were highly covered by the weed, we did not observe any significant change in the wild blueberry rhizosphere fungal and bacterial community. Although we took care sample wild blueberry rhizosphere for DNA extraction, it is not possible to exclude all weed roots during the sample preparation. Nonetheless, we were surprised to see a lack of difference in the microbial communities since weeds act as additional hosts for microbes and can, therefore, alter the microbial community (Schlatter et al., 2015). Nevertheless, de Vries and collaborators have argued that plant traits and edaphic conditions also have strong impacts on shaping the microbial community (de Vries et al., 2012). Soil pH, in particular, exerts a high selection pressure on the microbial communities (Fierer and Jackson, 2006; Rousk et al., 2010; de Vries et al., 2012).

The pseudo-replicate similarity analysis is interesting as we could have expected that fungal and bacterial communities samples from the same plot, sampled a dozen of centimeters apart, would be more similar than to the other samples. However, this is not what we have observed with our beta-diversity analyses ordinations, either relying solely on phylogenetic distance (unweighted Unifrac distance) or by sequence abundance (Aitchison distance). In both cases, the samples taken from the same plot do not group closer to each other than two other samples (Supplementary Figure 12). These results reinforce the fact that bacterial and fungal communities show a high diversity at small scales (Bach et al., 2018; Smercina et al., 2021) and that scientists should consider composite sampling, when it is feasible, to try to capture a broader scope and a better representativity of the microbial communities present in a given environment during the moment of sampling (Bullington et al., 2021).

### The fungal community is dominated by known and putative ericoid mycorrhizal taxa

Our analysis of the fungal community shows a predominance of the Helotiales order, totaling 244 ASVs and representing 46.8% of the relative abundance (RA). This fungal order contains known or putative ericoid mycorrhizae, most of which were identified in our dataset, including *Pezoloma ericae* (25 ASVs, 12.8% RA), *Oidiodendron maius* (15 ASVs, 9% RA), *O. chlamydosporicum* (2 AVS, 0.03% RA)*, O. tenuissimum* (1 ASV, 0.02% RA)*, Meliniomyces* sp. (19 ASVs, 1.7% RA), *Hyaloscypha variabilis* (12 ASVs, 2.6% RA), *H. bicolor* (1 ASV, >0.01% RA), *Lachnum pygmaum* (1 ASV, 0.08% RA), and *Mycosymbioces* sp. (11 ASVs, 7.2% RA) (Vohník et al., 2005, 2007; Grelet et al., 2009; Walker et al., 2011; Leopold, 2016; Fadaei, 2019). This order also contains the dark septate endophytes, *Phialocephala fortinii* (9 ASVs, 1.2% RA), and *P. glacialis* (5 ASVs, 0.5% RA), which may have possible beneficial outcomes on the plants they colonize (Newsham, 2011; Lukešová et al., 2015). Still, in the Ascomycota phylum, the Chaetothyriales order is the second most abundant with 59 ASVs and 28.6% RA, mainly composed of Herpotrichiellaceae (32 ASVs, 27.3% RA), a family often found in proximity with Ericaceae host plants (Midgley et al., 2004; Walker et al., 2011) and which contains the *Capronia* genus that was found to form hyphal coils in *Gaultheria shallon* roots (Allen et al., 2003). Switching to the less-abundant Basidiomycota phylum, the three orders, which stand out: Agaricales, Trechisporales, and Sebacinales, are also of interest. In our data, the Agaricales are dominated by *Clavaria sphagnicola* (9 ASVs, 3.8% RA), a genus known to form hyphal coils in Ericaceae roots (Peterson et al., 1980) and which is considered as a putative ericoid mycorrhizal fungal genus (Yang et al., 2018). *C. sphagnicola* is phylogenetically very close to *C. argillacea*, which was found making reciprocal exchanges of nutrients with rhododendron roots (Englander and Hull, 1980). The Trechisporales order is mainly composed by *Luellia sp*. (11 ASVs, 3.4% RA), a genus containing saprotrophic fungi with some putative ectomycorrhizal fungi (Malysheva et al., 2018). Although no ErM fungi have been identified in this genus, research has shown that ectomycorrhizal fungi can also colonize Ericaceae roots (Villarreal-Ruiz et al., 2004, 2012; Vrålstad, 2004). Furthermore, saprotrophic fungi can help degrade plant debris and extract nitrogen and phosphorus from lignocellulose more efficiently than mycorrhizal fungi and can, therefore, be beneficial for plant growth (Vohník et al., 2012). Finally, the Sebacinales order mainly composed of *Serendipita* sp. (13 ASVs, 0.9% RA) was identified as having potential ericoid mycorrhizal fungi species (Vohník et al., 2016). Overall, our data show a relatively high abundance of symbiotic and/or endophytic fungal taxa in the rhizosphere community, which may suggest their importance in the wild blueberry rhizosphere ecosystem. The confidence in our taxonomy annotation is reinforced with the mock community analysis, which correctly assigned the correct genus to 17 on 19 taxa, and 10 to the species level (Supplementary Information). However, although we used an even mock community, where each taxon should be represented equally, we witnessed a higher abundance of Ascomycota taxa than Basidiomycota in our mock community results. While Ascomycota have been reported to dominate the wild blueberry rhizosphere (Yurgel et al., 2017; Morvan et al., 2020), the relative abundance obtained in our mock and experimental communities could be skewed as either the PCR amplification or sequencing could have introduced a bias toward Ascomycota over Basidiomycota sequences.

### The bacterial community contains abundant taxa with carbon-degrading capacity, as well as dinitrogen fixation potential taxa

The bacterial dataset was dominated by Frankiales (175 ASVs, 14% RA), mostly belonging to the *Acidothermus* genus (134 ASVs, 12.9% RA). To date, this genus has had a sole known species: *A. cellulolyticus* isolated from acidic hot spring in Yellowstone National Park. As the name of the genus implies, the bacteria are acidophilic (optimal pH = 5) and thermophilic (optimal temperature = 55°C) (Mohagheghi et al., 1986). Both the acidophilic and thermophilic natures of the bacteria are coherent in a wild blueberry context, as temperature records show that soil in the blueberry field (in which this experiment is part of) often reaches 40°C, with a max temperature of 53.07°C in the summer 2021. Furthermore, *A. cellulolyticus* genome sequence contains numerous plant biomass and fungal cell wall-degrading enzymes (Barabote et al., 2009), a characteristic consistent in a wild blueberry soil, which contains a high level of organic matter. Among the Acidobacteriales (236 ASVs, 12.5% RA), most belonged to the Acidobacteriaceae Subgroup 1 (148 ASVs, 6.2% RA), which was found to be abundant in low-pH soils and is able to degrade a variety of carbon sources (Kielak et al., 2016). The Isosphaerales order (209 ASVs, 12.4% RA) commonly found in acidic northern wetlands (Kulichevskaya et al., 2016) was dominated by the *Aquispharea* genus (81 ASVs, 10.1% RA), first identified from a freshwater aquarium (Bondoso et al., 2011). Although *Aquispharea* was not included in the study, Ivanova and collaborators have identified a common pool of carbohydrate-active enzymes in four other Isosphaeraceae species, suggesting a high potential of this bacterial family to use a variety of carbohydrates and glycoconjugates (Ivanova et al., 2017). The Rhizobiales order (150 ASVs, 10.4% RA), well-known to harbor nitrogen-fixing bacteria, was dominated by the Xanthobacteraceae family (82 ASVs, 7.3% RA) and the *Roseiarcus* genus (31 ASVs, 2.5% RA). Xanthobacteraceae contain dinitrogen fixation potential (Sawada et al., 2003; Oren, 2014), among which *Rhodoplanes* (Buckley et al., 2007) and *Bradyrhizobium* (Ormeno-Orrillo and Martinez-Romero, 2019) have been previously reported to fix dinitrogen and are also present in our community. *Roseiarcus fermentans*, the only known species of this genus to date, was isolated from acidic Sphagnum peat and is capable of dinitrogen fixation. This genus was also found to be abundant in a previous study on wild blueberry rhizosphere (Morvan et al., 2020). Our bacterial mock community analysis supports our taxonomy assignment as 18 out of the 20 species present in the mock community were correctly assigned to the species level, and the two remaining had a correct genus identity. Regarding the abundance, we observed no bias toward a particular taxonomy level. Regrettably, there were no Acidobacteriota nor Planctomycetota taxa in the mock community that was sequenced, while they represent a significant proportion of the community found in our experiment.

## Conclusion

This study aimed to evaluate the impact of thermal pruning on agronomic variables and the microbiome of wild blueberry in an agricultural setting. Our results show limited, short-term benefits of burning. *Septoria* leaf spot disease was negatively impacted by burning at a high intensity in the short term. However, this temporary reduction had no effect on blueberry yield. We observed a tentative non-significant decrease of the predominant weed, *Cornus canadensis*, due to burning. Since *C. canadensis* is a common wild blueberry competitor, thermal pruning could be worthwhile, but additional experiments are required to validate our results as they are in contradiction with the literature. Moreover, the burning intensities used in our experiment did not significantly disrupt the fungal and bacterial communities of the wild blueberry rhizosphere sampled during the subsequent harvesting year (15 months after the thermal pruning treatment). Otherwise, as we did not record any other significant beneficial outcome, thermal pruning may not be worth investing into. The use of alternative technics like vinegar as a herbicide (practice already used in cranberry fields) or products containing *Bacillus subtilis* as fungicides are more promising and ecofriendly than burning fossil fuels. This aspect should be taken into consideration, especially during our times of climatic crisis.

## Data availability statement

The agricultural datasets for this study can be found in Zenodo: https://doi.org/10.5281/zenodo.6544474. The R scripts used to analyze the data and to generate figures are deposited on GitHub: https://github.com/SimonMorvan/thermal_BB_microbiome. The microbial sequences have been deposited in the NCBI GenBank Sequence Read Archive under the accession number PRJNA803472: https://www.ncbi.nlm.nih.gov/bioproject/PRJNA803472.

## Author contributions

MP and JL conceived and designed the agronomic part of the study, while MH and SM conceived and designed the microbial part of the study. AS and SM contributed to data acquisition. SM performed the microbial community analysis experiments, analyzed the data, and wrote the first draft of the manuscript. All authors reviewed and discussed the results, read, and approved the submitted version of the article.

## Funding

This study received funding from the following source: the Natural Sciences and Engineering Research Council of Canada (NSERC) Discovery grant (Grant RGPIN-2018-04178) to MH ; the Syndicat des Producteurs de Bleuets du Québec (SPBQ) and the Natural Sciences and Engineering Research Council of Canada (NSERC) (Grant RDCPJ-503182-16) to MP.

## Conflict of interest

The authors declare that the research was conducted in the absence of any commercial or financial relationships that could be construed as a potential conflict of interest.

## Publisher's note

All claims expressed in this article are solely those of the authors and do not necessarily represent those of their affiliated organizations, or those of the publisher, the editors and the reviewers. Any product that may be evaluated in this article, or claim that may be made by its manufacturer, is not guaranteed or endorsed by the publisher.
