# Supplementary material for: Limited effect of thermal pruning on wild blueberry crop and its root-associated microbiota
Source: Front Plant Sci. 2022 Aug 4;13:954935. doi: 10.3389/fpls.2022.954935 (PMC9408806; doi:10.3389/fpls.2022.954935)
Supplement: Supplementary file 2 [file Data_Sheet_2.docx]

**Supplementary methods**

**16S PCR workflow**

**ITS PCR workflow**

**Indexation PCR workflow**

**Supplementary information**

Indicative species analysis

Complementary to the Metacoder analysis, we proceeded with an indicative species analysis. This method identifies specific species that are representative of pre-defined groups of samples based on the species abundance and fidelity. In our case, we used phyloseq’s tax_glom function to agglomerate the ASVs at species level and set the pre-defined groups as the four burning intensities. We used the *multipatt()* function of the indicspecies R package ([de Cáceres and Legendre, 2009](#_ENREF_22)). Each ASV is assigned an indicative species value (IndVal) based on its importance in the group it is found to be most indicative of. A second analysis allowed for relevant site combinations (example: highest with medium burning intensity but not highest and negative control) by setting option duleg=False and specifying which group combinations were relevant. For both analysis, with or without group combinations, 9999 permutations were used to compute the p values of each IndVal which were corrected with an FDR method to account for multiple comparisons.

de Cáceres, M., and Legendre, P. (2009). Associations between species and groups of sites: indices and statistical inference. *Ecology* 90(12)**,** 3566-3574.

Data processing

After processing the sequences through the bio-informatic pipeline, we obtained 980 353 ITS sequences (30 636 ± 7 110 reads on average per sample) dispatched in 913 ASVs (159 ± 34 on average per sample) and 738 599 16S reads (23 081 ± 6114 reads on average per sample) dispatched in 4971 ASVs (1294 ± 132 on average per sample). Comparing the taxonomy assignment obtained using the UNITE fungal reference database and eukaryotic reference database allowed to remove 106 ASVs that were labelled with a non-fungal Kingdom, most of which (81) were labelled as Viridiplantae belonging to Ericaceae species**.** Singleton and doubleton removal eliminated an additional 45 ASVs. In the bacterial dataset, we removed 12 Archaea, 22 Chloroplasts and 11 Mitochondria ASVs as well as 261 singletons and 615 doubletons. Finally, to evaluate if we had any contamination, we searched for common ASVs between our samples and both the extraction and PCR blanks. Regarding the PCR blanks, none of the sequences originally present in the PCR blank made it through the fungal pipeline while one ASV with 52 reads was obtained in the bacterial dataset and was unique to the 16S PCR blank sample. For the extraction blanks, in the fungal dataset, we obtained 15 170 sequences dispatched in 4 ASVs. One of these ASVs was also present in 21 out of 32 samples totaling 437 reads. Out of precaution, we removed this ASV identified as *Troposporella monospora* from our sample dataset. For the bacterial dataset, the extraction blank resulted in 12438 reads dispatched in into 18 ASVs. Seven of those 18 ASVs were also found in our sample dataset and were removed from the bacterial sample dataset as they could be potential contaminants. These extra refining steps resulted in a total of 768 fungal ASVs and 4016 bacterial ASVs in our samples. The rarefaction curves obtained for both bacterial and fungal datasets indicate a sufficient sequencing depth (Fig. S4).

Mock community analysis

The fungal mock community contained 19 species belonging to the Ascomycota (12), Basidiomycota (3), Mucoromycota (2), Glomeromycota (1) and Chytridiomycota (1). Two species present in the mock community weren’t found in our sequenced mock, *Rhizophagus irregularis* (Glomeromycota) and *Rhizomucor miehei* (Mucoromycota). The other 17 species were either correctly identified in our sequences mock (10) or had at least the same genus (7). One of the species, *Naganishia albida* (Basidiomycota) was separated into two ASVs in our sequenced mock. Although the mock community was conceived to contain even concentration of DNA material of each species, we observe a higher number of sequences for Ascomycota sequences than the rest of the fungal phyla which could indicate an amplification or sequencing bias (Table S2). The bacterial mock community contained 20 species with equimolar counts (10^6^ copies/μL) of 16S rRNA genes. Out of the 20 species, 18 were identified to species level in the sequenced mock community. Our pipeline failed to assign a species to the *Actinomyces* sp. sequence and *Bacillus cereus* was identified as *Bacillus anthracis*. Furthermore, there were additional taxa found in that aren’t listed in the mock community: *Staphylococcus caprae* (640 reads), *Lactobacillus paragasseri* (2 reads), Chloroplast (O, 12 reads), Mitochondria (F, 9 reads), and Xanthobacteraceae (F, 3 reads). The pipeline also found multiple ASVs assigned to a similar species with 6 *Bacteroides vulgatus* ASVs and 2 *Clostridium beijerinckii* ASVs (Table S3).

**Supplementary figures**

**Figure S1**

Experimental design, visual aspect of the treatment and soil temperature profile measured during the burning treatment. A. and B. illustrate the experimental design. C**.** show the pruning treatment in action. D. displays the 1 cm depth temperature profile measured during the burning treatments.

A.


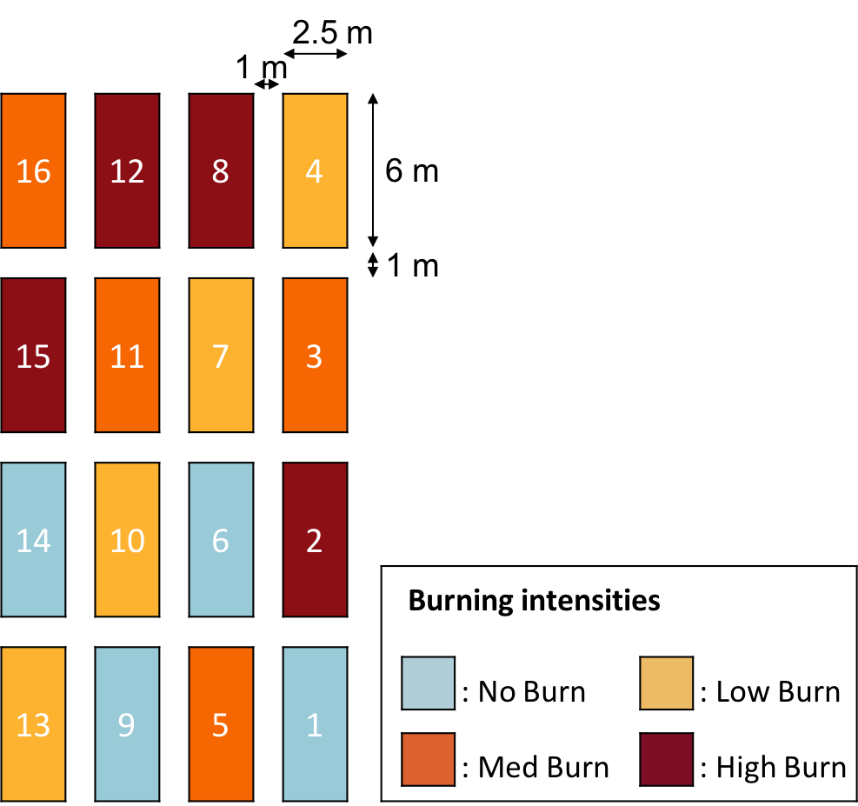


B.


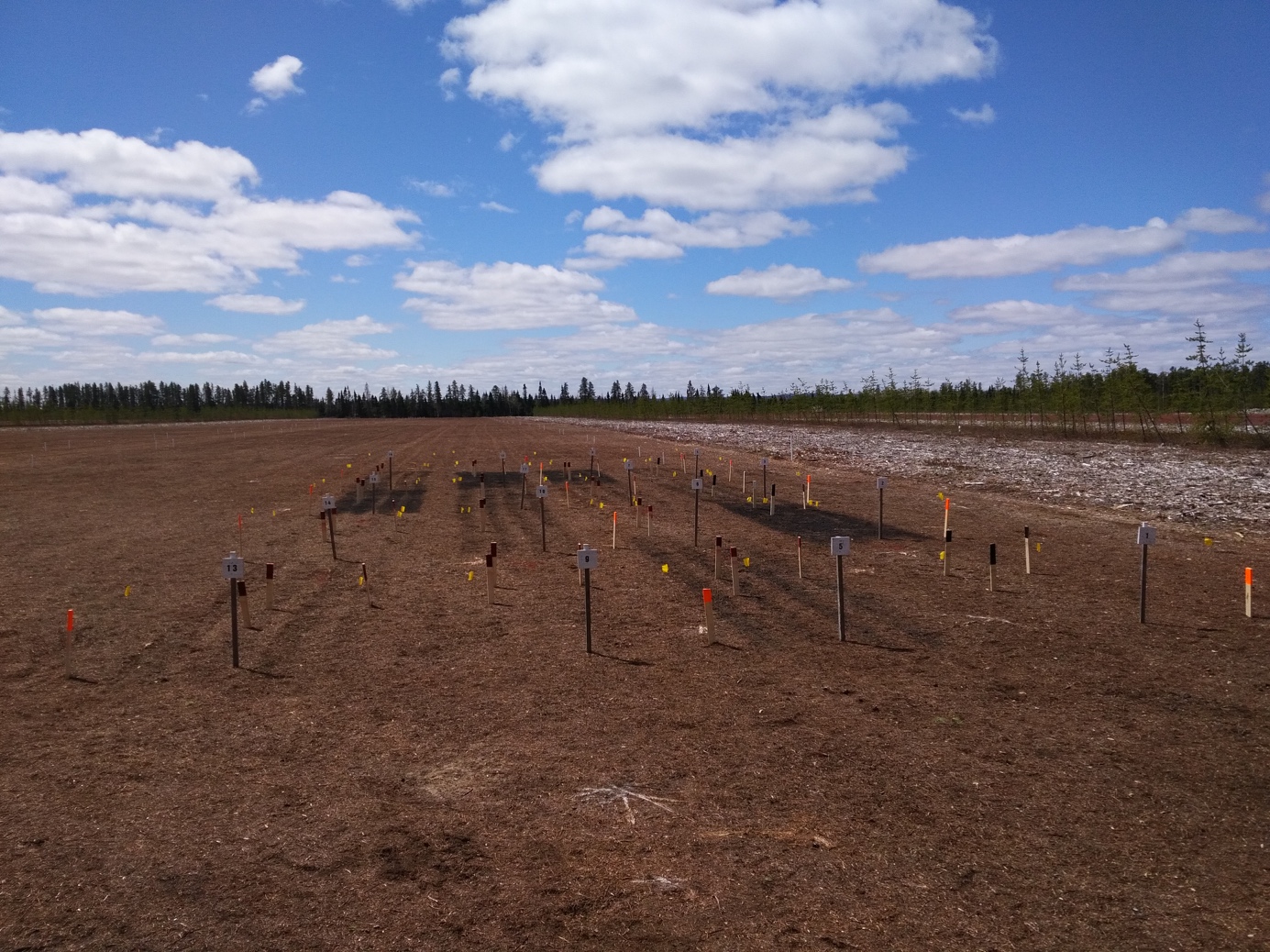


C.


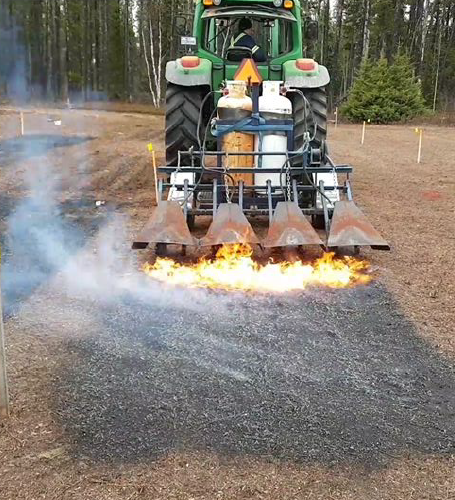


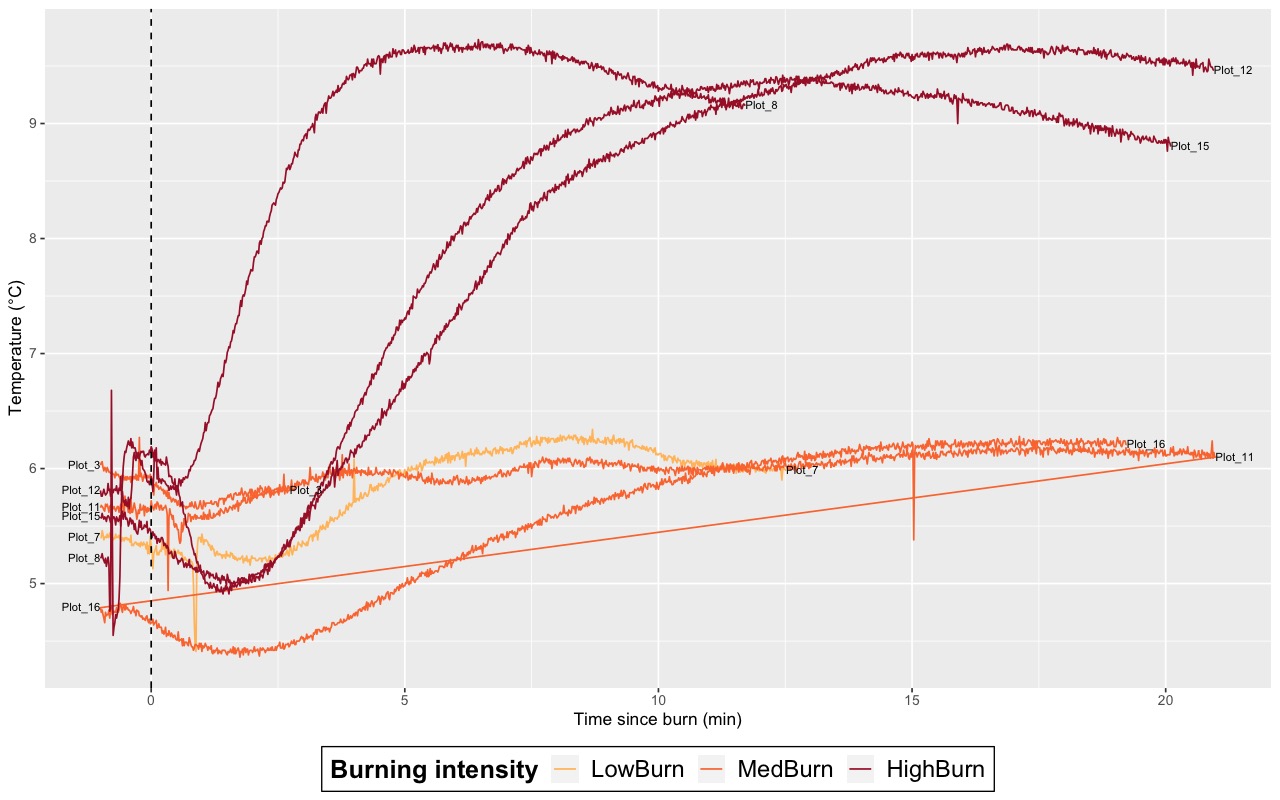
D.

**Figure S2**

Illustration of sample preparation for DNA extraction.


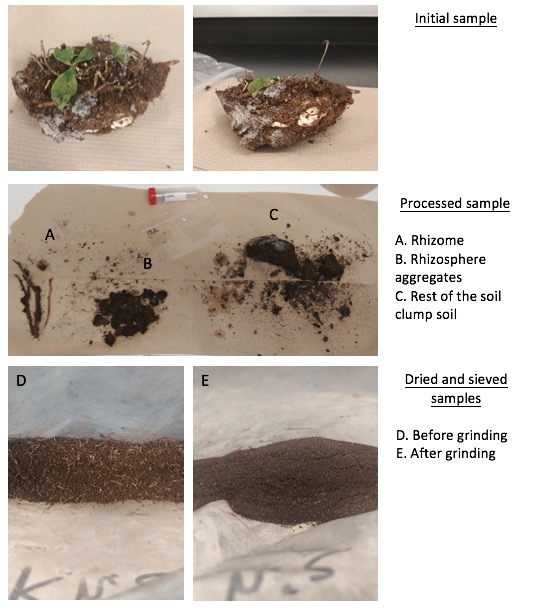


**Figure S3. Rarefaction curves**

1.
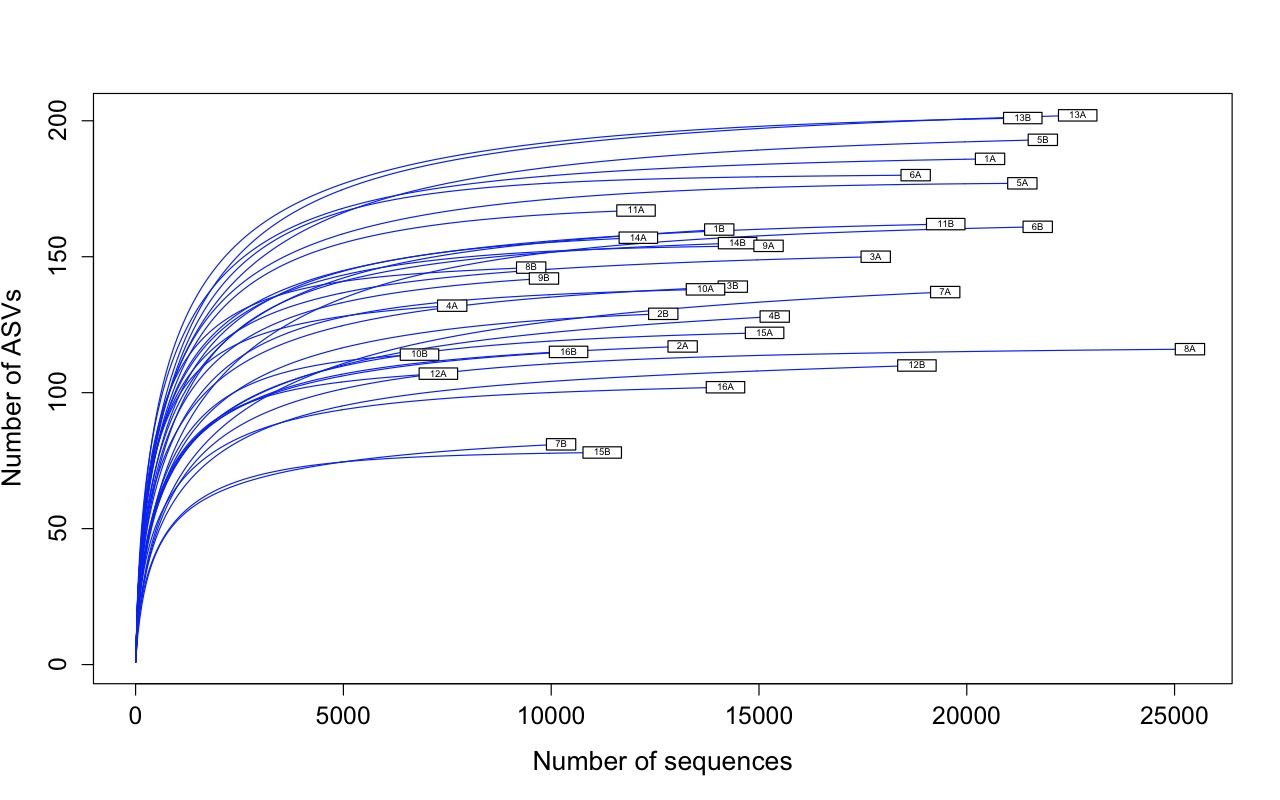
Rarefaction curve for the fungal community.
2.
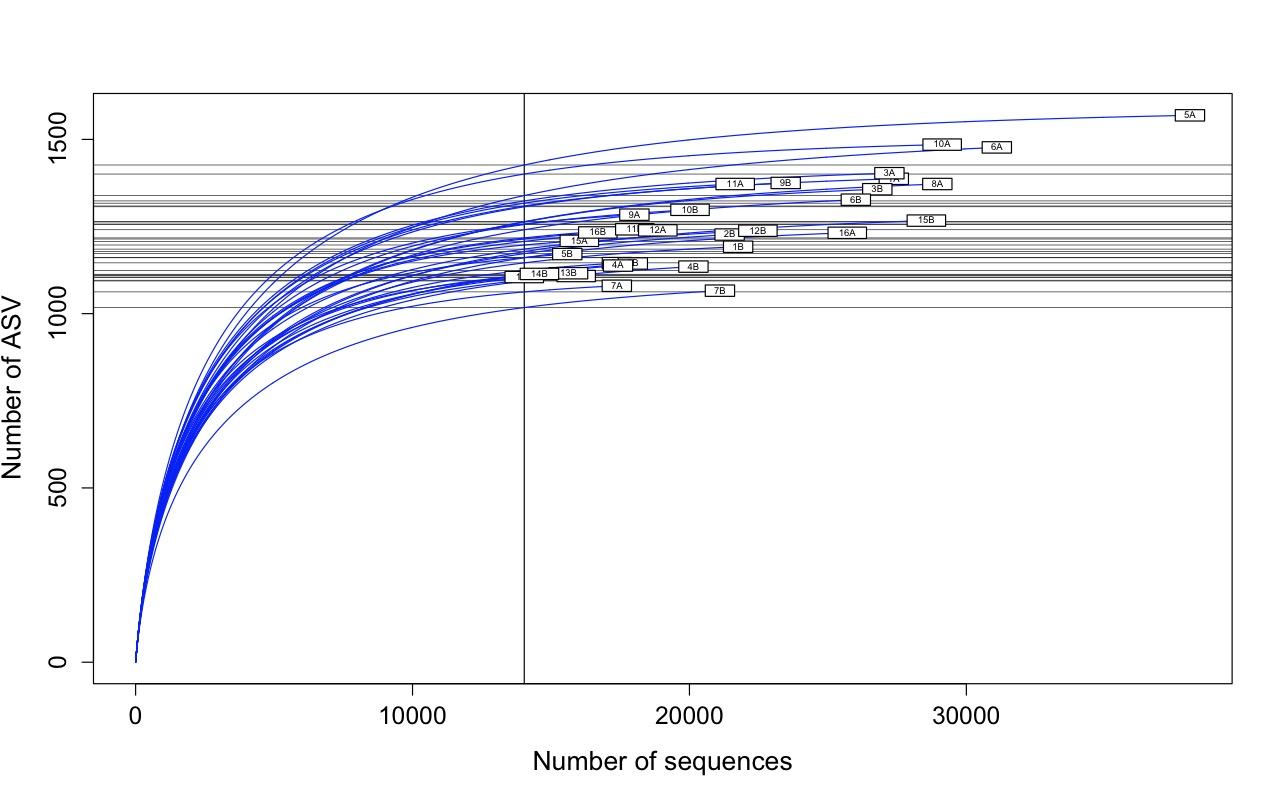
Rarefaction curve for the bacterial community.

**Figure S4. Effect of burning on soil organic layer thickness (A) and humidity content (B)**

Mean value is indicated with a black circled dot. P values computed by ANOVA are mentioned in each facet.

**
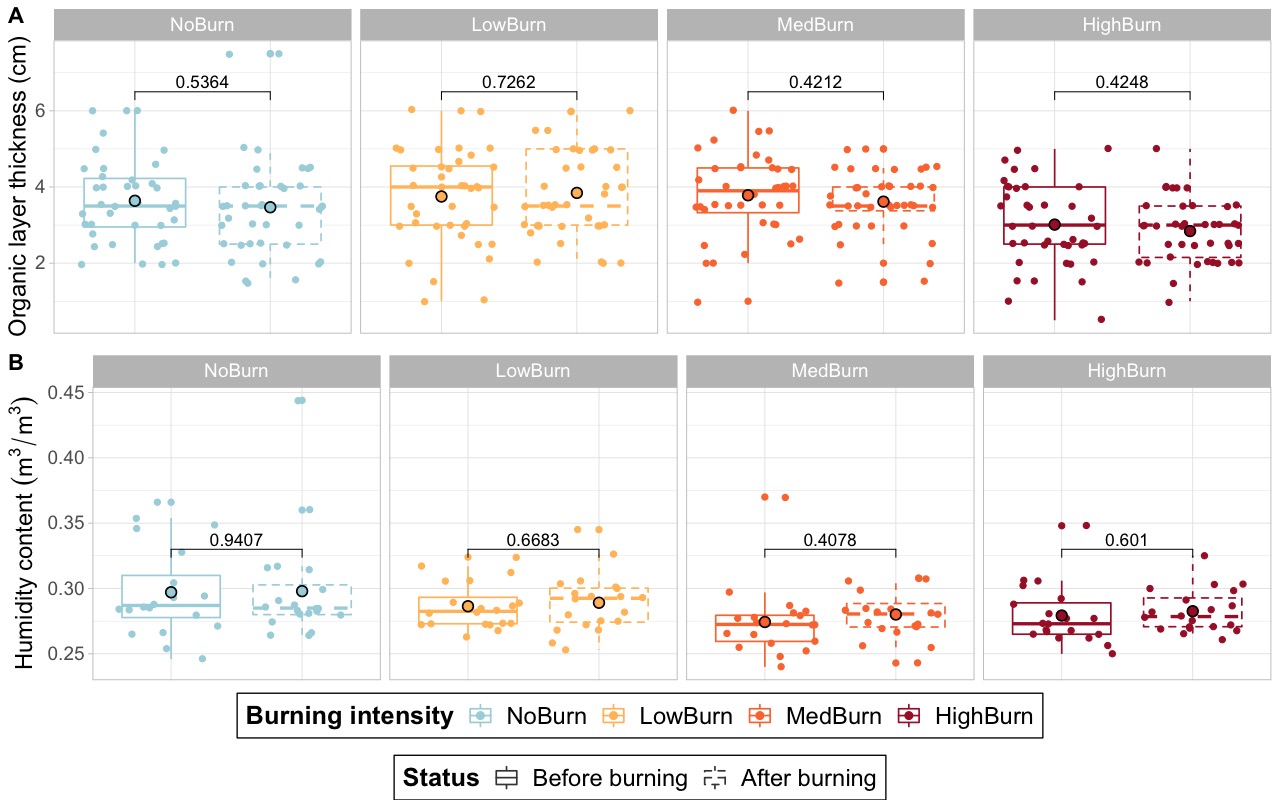
**

**Figure S5. Soil pH measurements over time.**

No significant differences observed. Mean value is indicated black circled dot. (A) Organic layer. (B) Mineral layer.

**
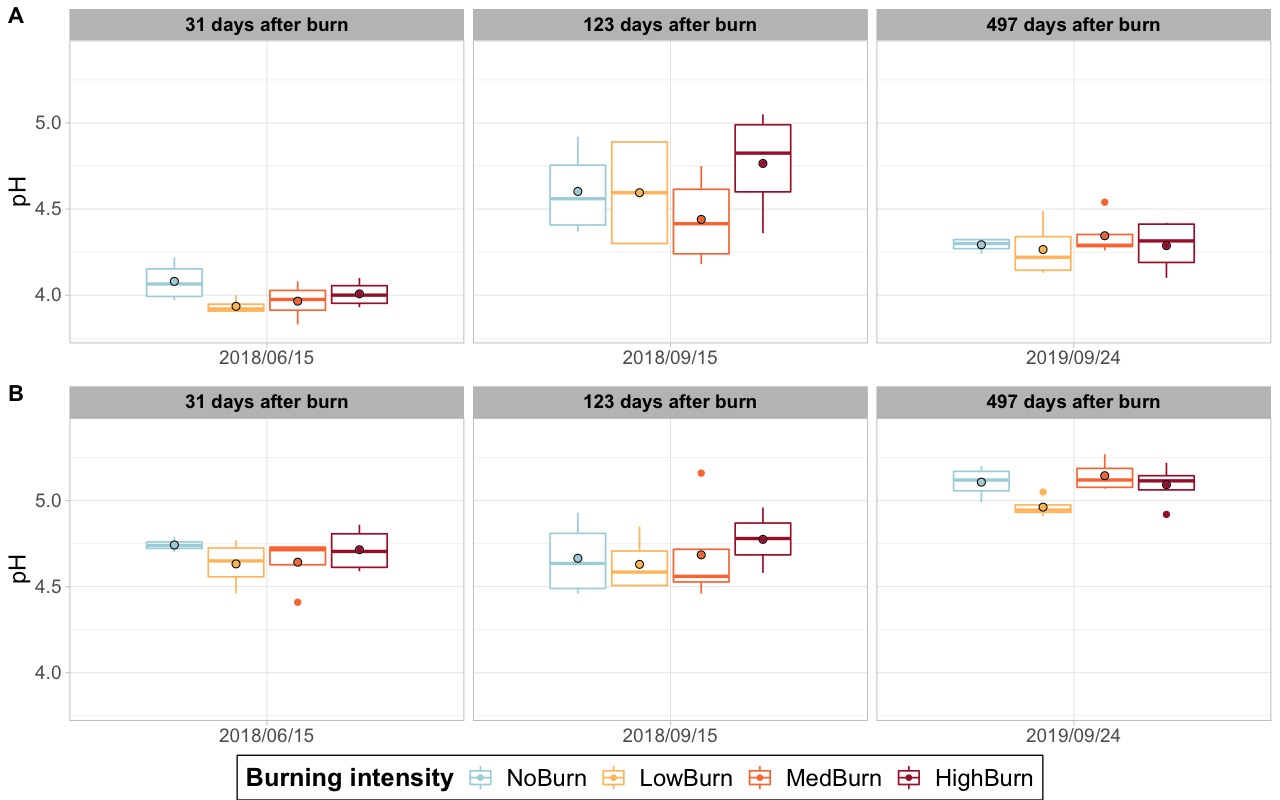
**

**Figure S6. Soil carbon concentration over time.**

No significant differences observed. Mean value is indicated black circled dot. (A) Organic layer. (B) Mineral layer.

**
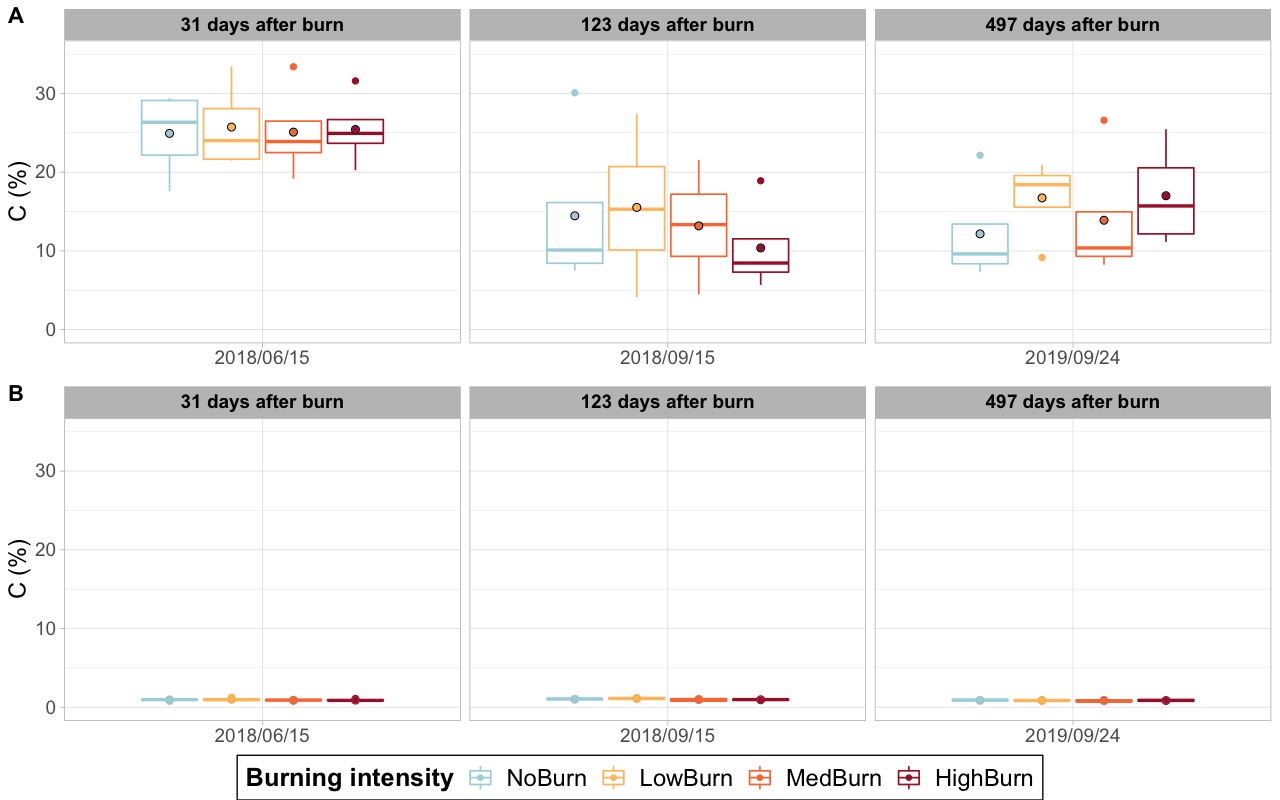
**

**Figure S7. Soil nitrogen concentration over time.**

No significant differences observed. Mean value is indicated black circled dot. (A) Organic layer. (B) Mineral layer.


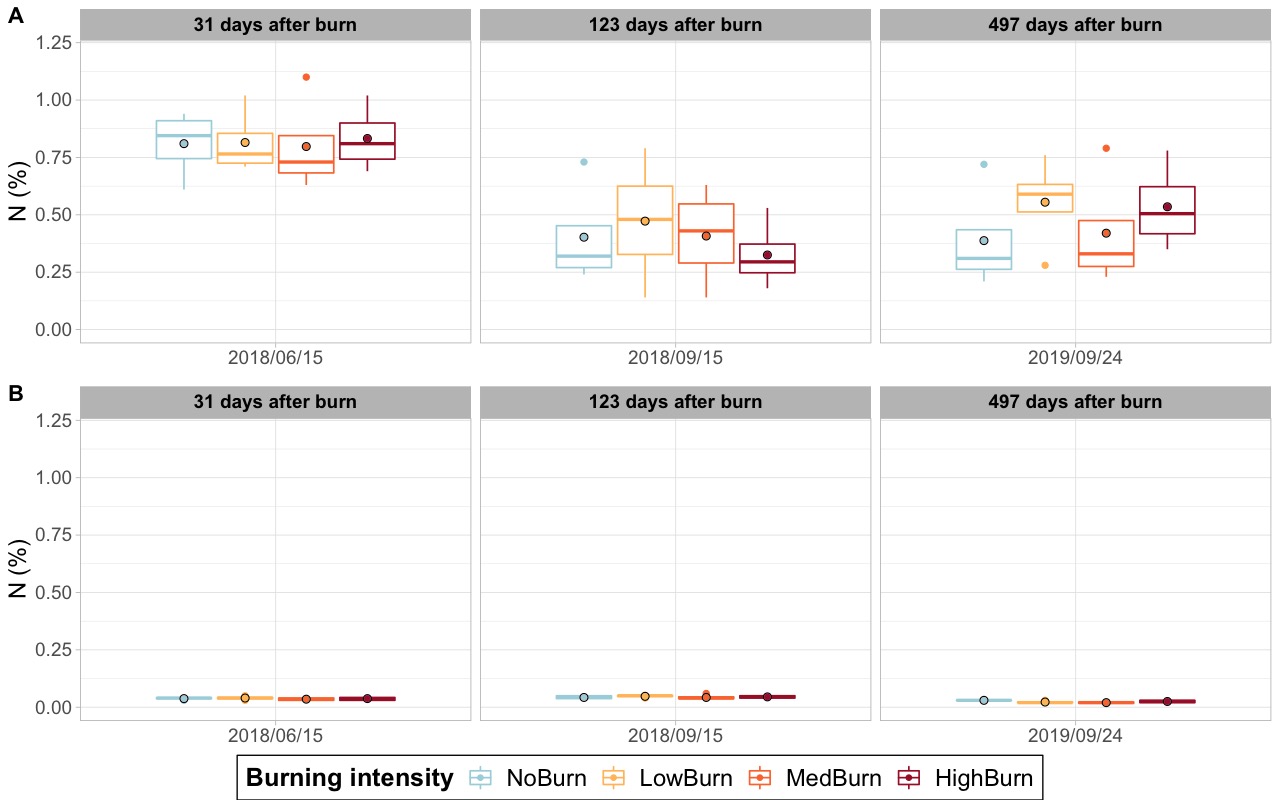


**Figure S8. Soil potassium concentration over time.**

Significant difference in each sampling date is indicated by letters according to post-hoc Tukey tests. Mean value is indicated black circled dot. (A) Organic layer. (B) Mineral layer.


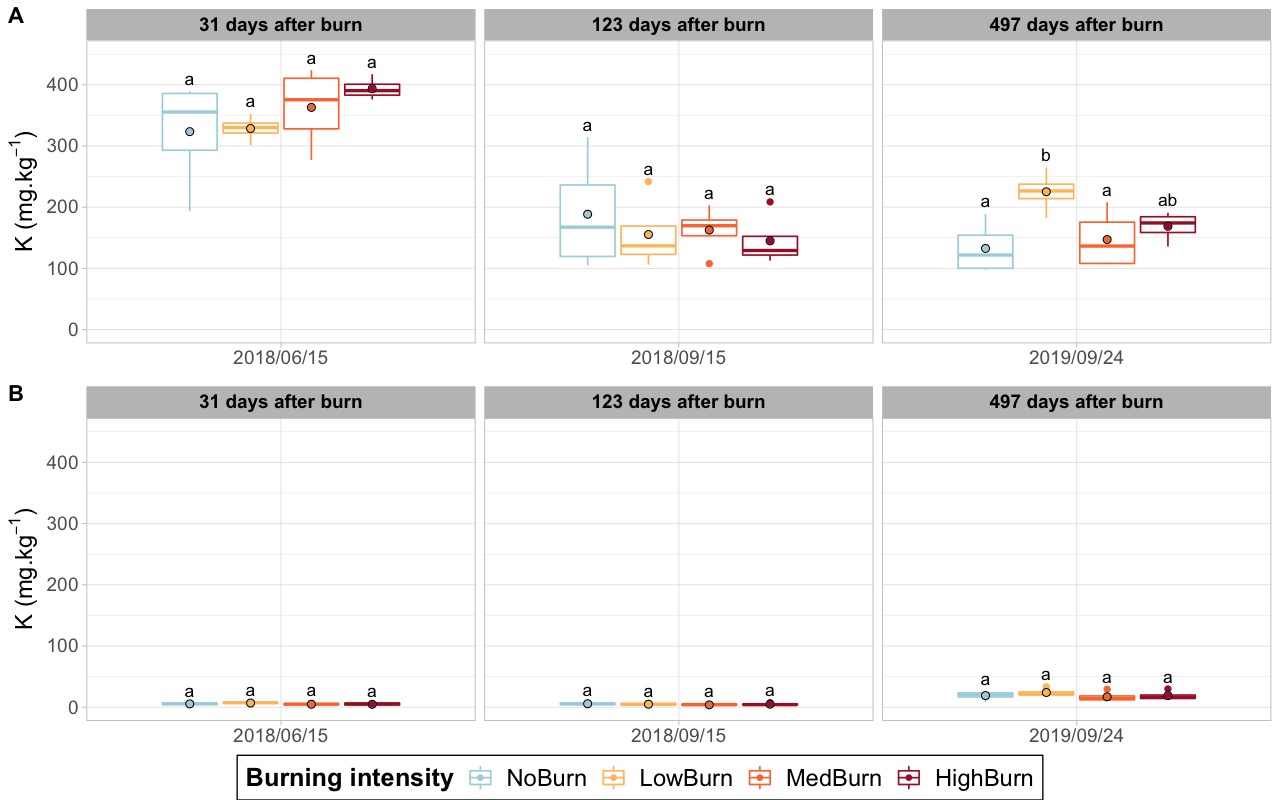


**Figure S9. Soil magnesium concentration over time.**

Significant difference in each sampling date is indicated by letters according to post-hoc Tukey tests. Mean value is indicated black circled dot. (A) Organic layer. (B) Mineral layer.

**
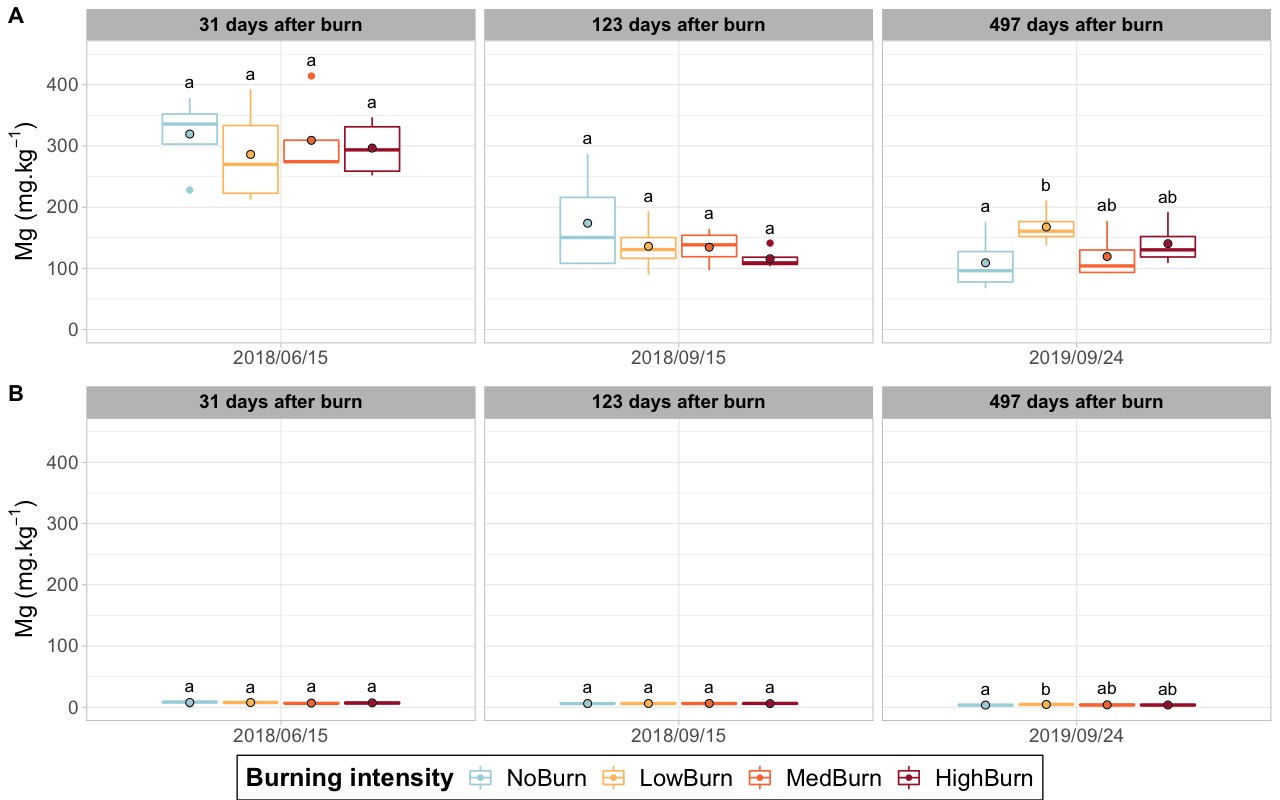
**

**Figure S10. Soil calcium concentration over time.**

Significant difference in each sampling date is indicated by letters according to post-hoc Tukey tests. Mean value is indicated black circled dot. (A) Organic layer. (B) Mineral layer.

**
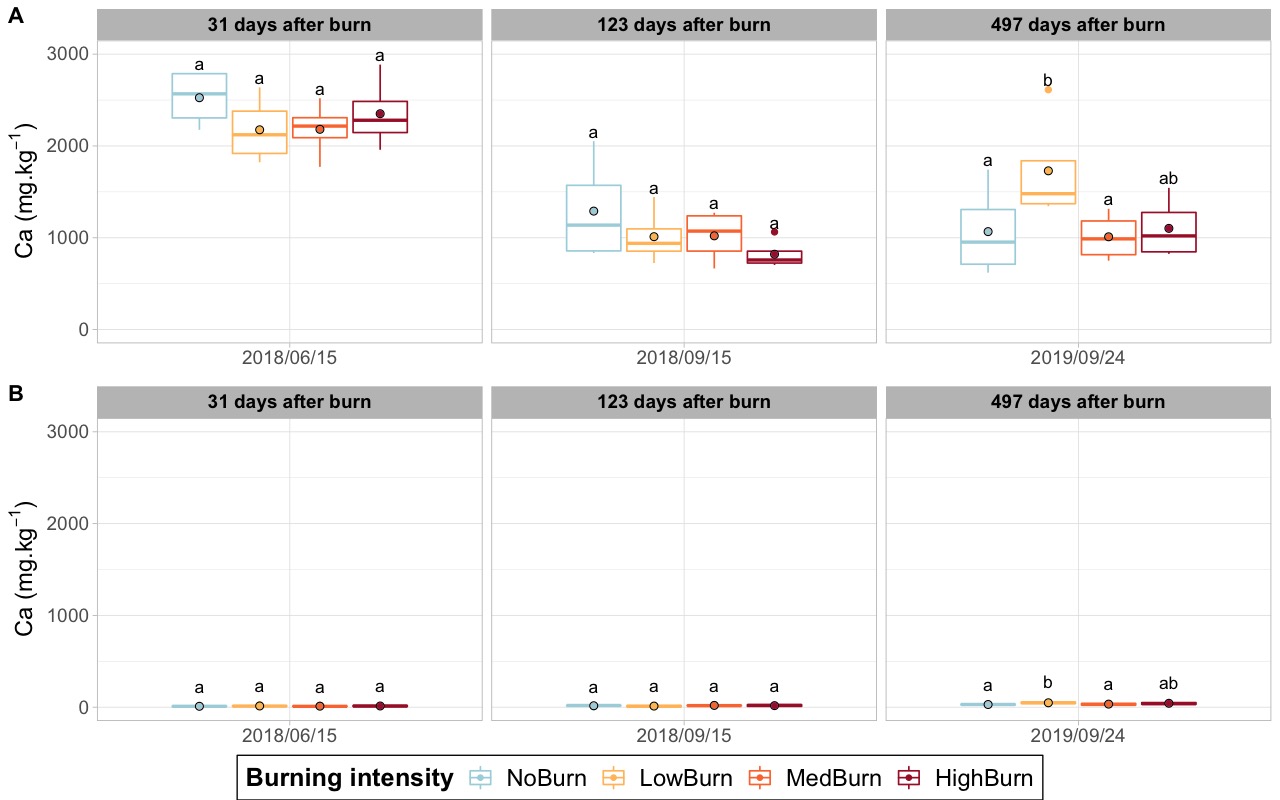
**

**Figure S11.** **Core microbiome** **of the fungal community (A) and the bacterial community (B).**

Each section contains the number of ASV common to the overlap of the group as well as the relative abundance (in parenthesis) these ASVs represent in the dataset.


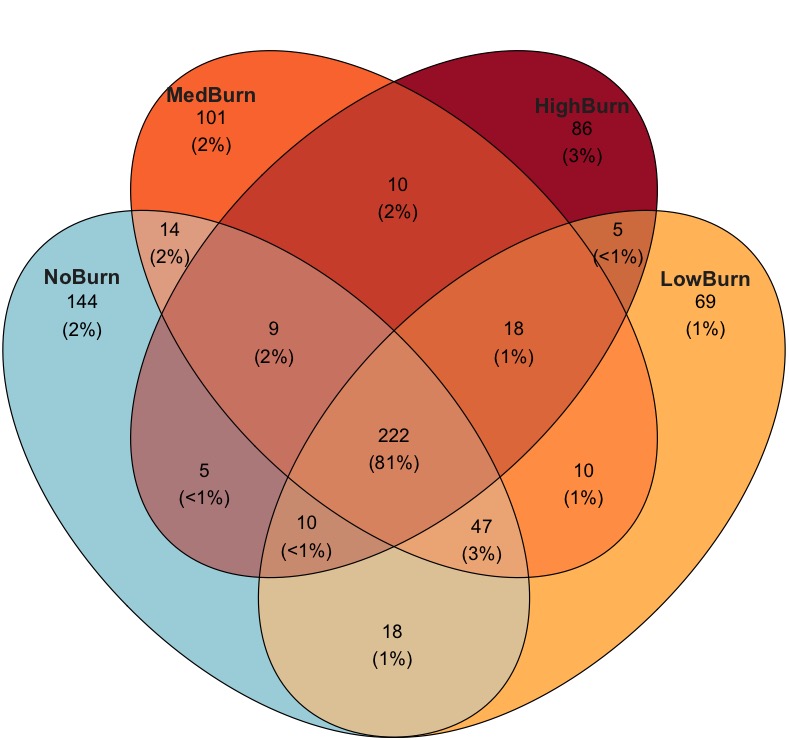


**
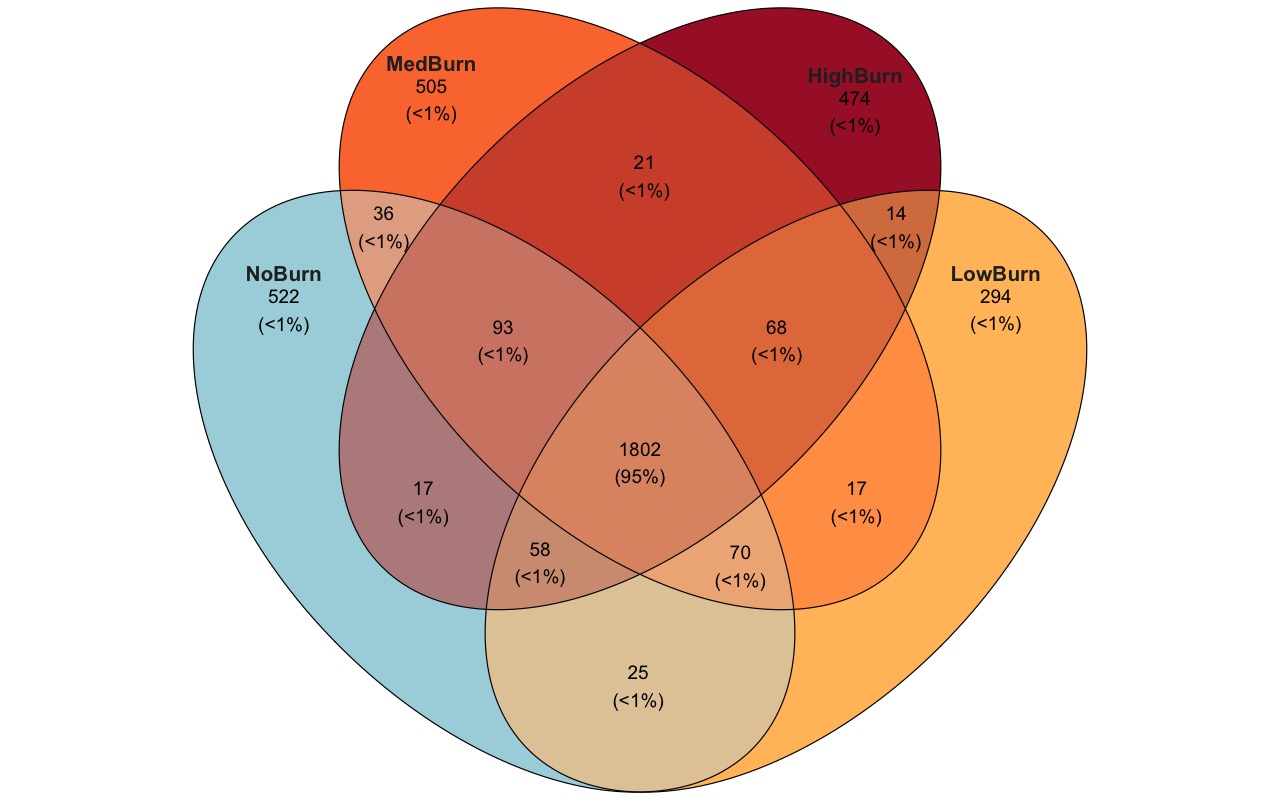
**

**Figure S12. Beta diversity of pseudo-replicate using the Aitchison and the unweighted Unifrac distances.**

A. Fungal community, B. Bacterial community.

A.

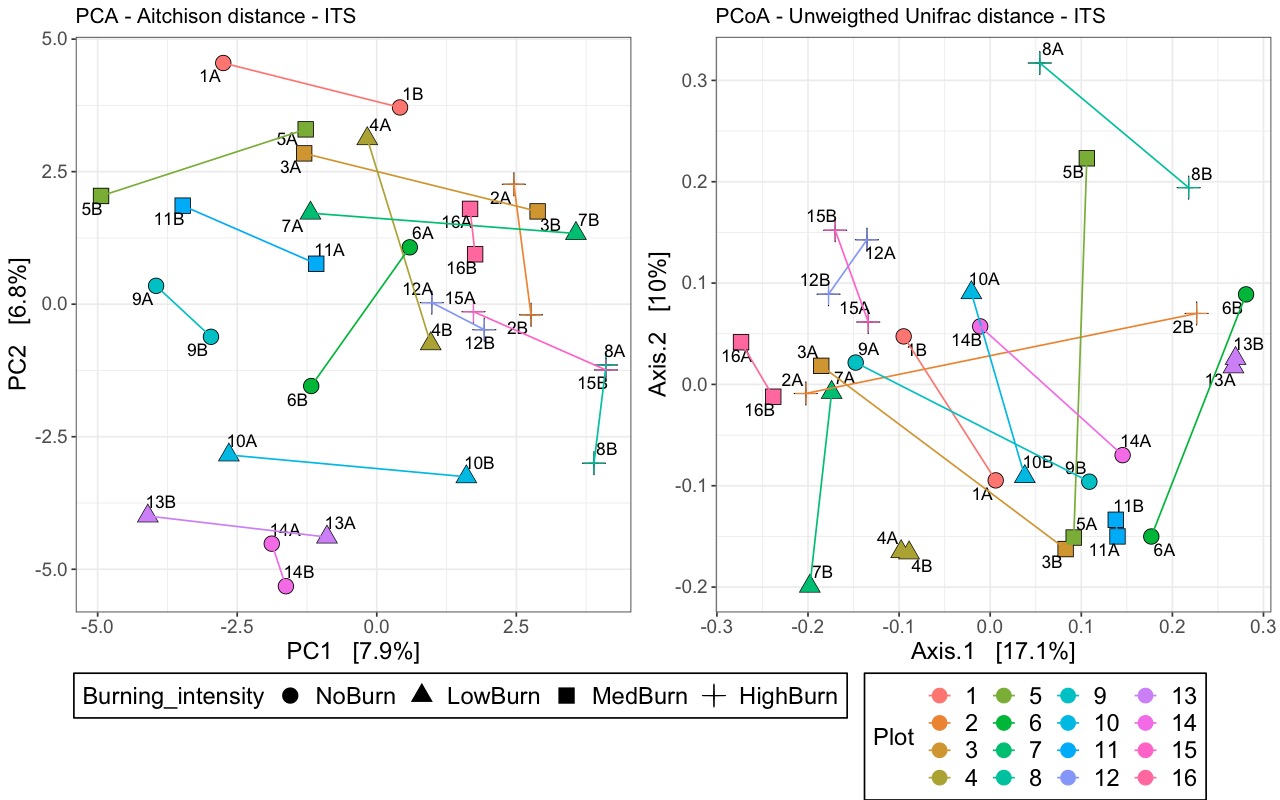


B.


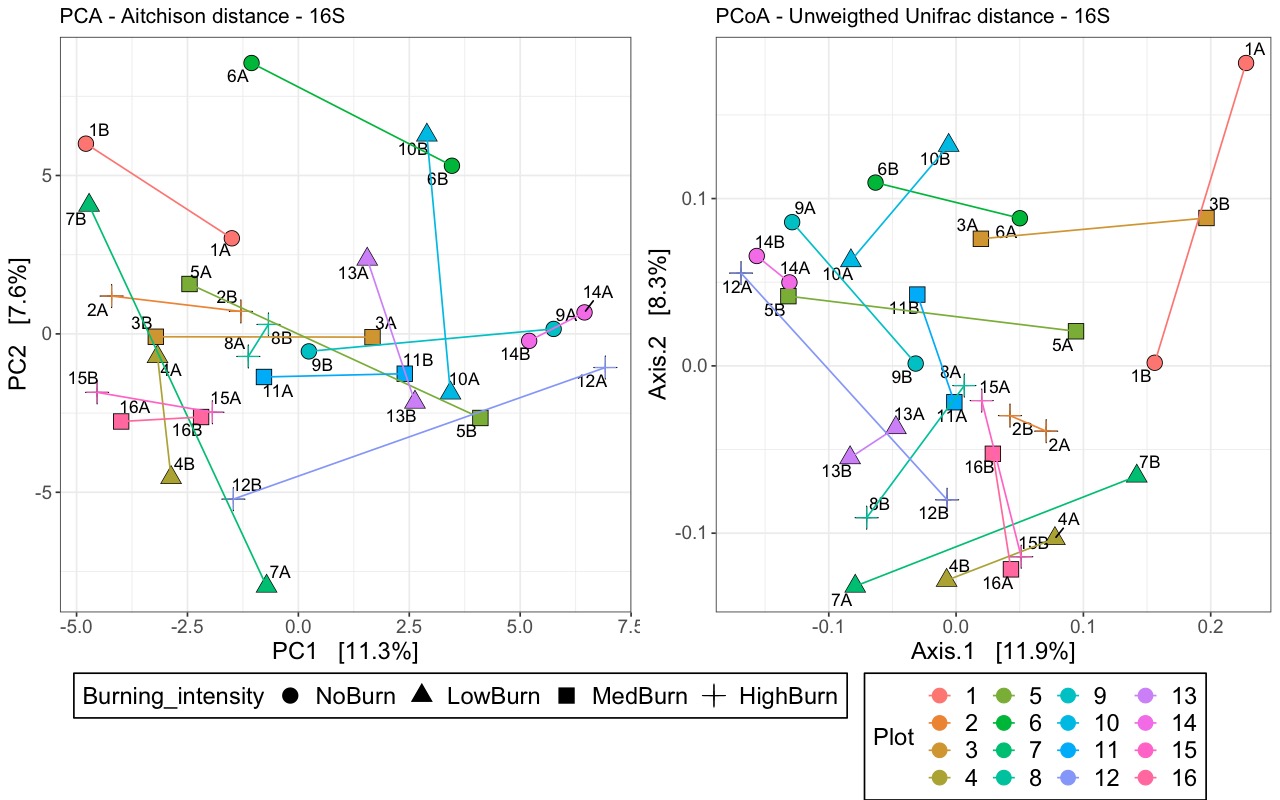


**Figure S13. Alpha diversity using Shannon-Weaver and Simpson reciprocal indices.**

A. Fungal community, B. Bacterial community.


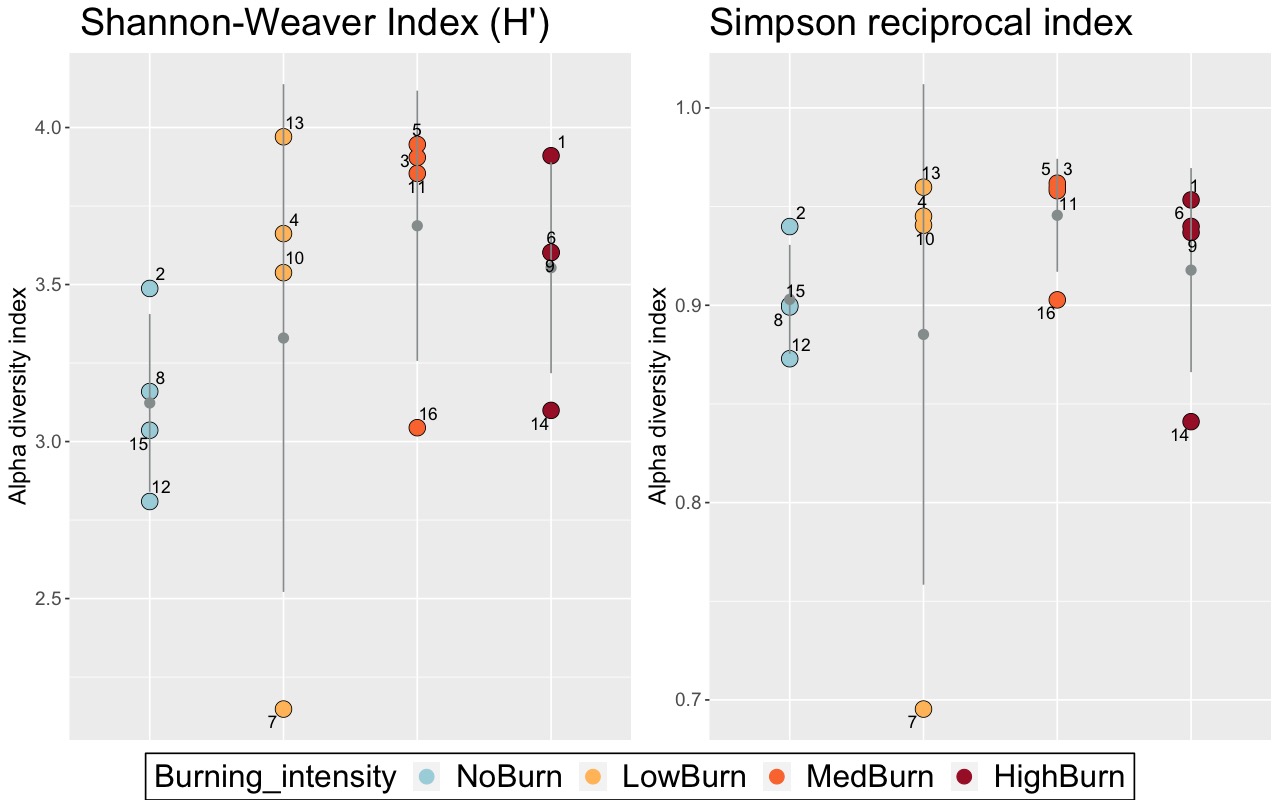


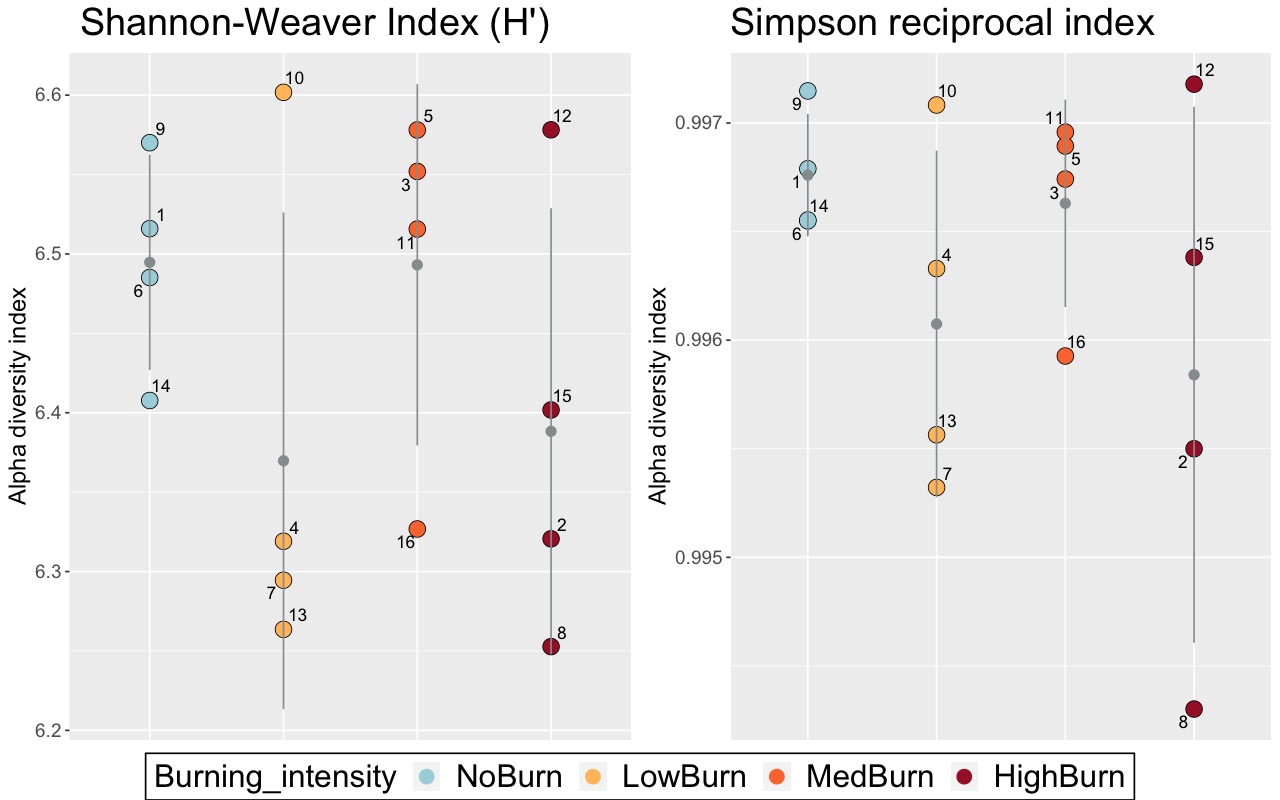


**
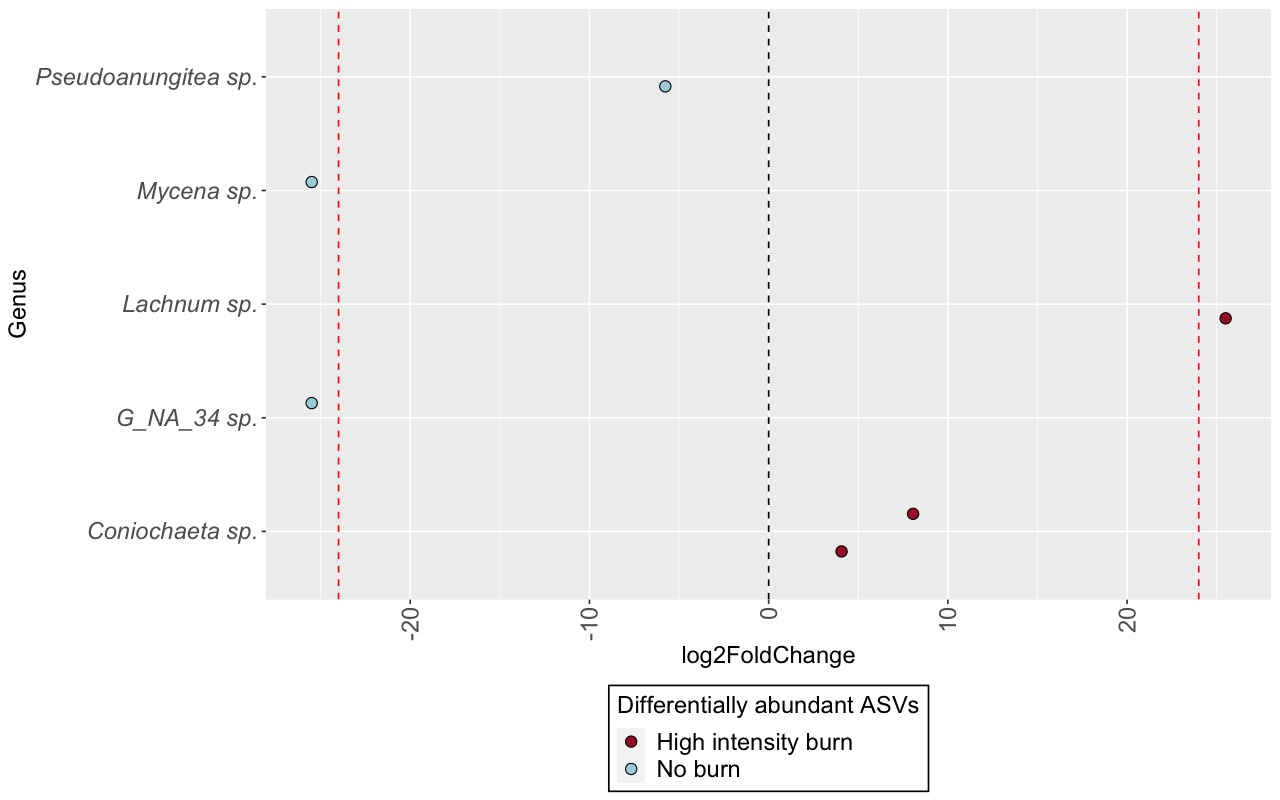
Figure S14**. **Differential abundance of fungal ASVs between the negative control and the highest burning intensity computed with DESeq2.**
